# Synergistic multifunctional biomedical activity of microwave-synthesized Au/Pt/Cu trimetallic nanofluids: experimental and DFT insights

**DOI:** 10.1039/d6ra06054d

**Published:** 2026-10-09

**Authors:** Navneet Yadav, Amara Rafi, Lalan Kumar, Binesh Kumar, Sikandar Paswan, Ajit Kumar Maddheshiya, Jai Devi, Nikunj Patel, Afsanehsadat Larimi, Bharat Kumar

**Affiliations:** a Department of Physics, University of Allahabad Prayagraj-211002 Uttar Pradesh India Navneet.yadav@swansea.ac.uk navaneetyadav840@gmail.com; b Department of Chemistry, Swansea University Singleton Park Campus Swansea SA2 8PP UK lalan.kumar@swansea.ac.uk; c Center of Agriculture, Food, Environment, University of Trento San Michele all’Adige 38010 Italy; d Department of Chemistry, Guru Jambheshwar University of Science and Technology Hisar-125001 India; e Department of Chemistry, Baba Raghav Das Post Graduate College Deoria-274001 Uttar Pradesh India; f Institute of Pharmaceutical Science, King's College London Franklin-Wilkins Building, Stamford Street SE1 9NH London UK; g Department of Chemical Engineering, Swansea University Bay Campus Swansea SA1 8EN UK; h Department of Chemistry, University of Lucknow Lucknow-226007 Uttar Pradesh India kumar_bharat@lkouniv.ac.in

## Abstract

In the present study, a trimetallic Au/Pt/Cu nanofluid was synthesized *via* a rapid one-pot microwave-assisted strategy and systematically evaluated for multifunctional biomedical activity. Structural and spectroscopic characterization confirmed the formation of an integrated multimetallic nanostructure, likely to contain alloyed or compositionally mixed domains that generate synergistically active interfaces of Au, Pt and Cu. The nanofluid exhibited broad-spectrum antimicrobial activity, inhibiting Gram-positive bacteria (*Staphylococcus aureus*, *Bacillus subtilis*) and Gram-negative bacteria (*Escherichia coli*, *Pseudomonas aeruginosa*) with a minimum inhibitory concentration (MIC) of 0.0137 µmol mL^−1^. Enhanced antifungal efficacy was observed against *Rhizopus oryzae* and *Candida albicans* (MIC = 0.0068 µmol mL^−1^), with activity against *C. albicans* surpassing that of fluconazole under identical conditions. The anti-inflammatory potential evaluated through bovine serum albumin denaturation revealed an IC_50_ of 14.88 ± 0.09 µM, while antioxidant capacity measured using the DPPH assay showed an IC_50_ of 4.34 ± 0.08 µM, indicating effective free-radical scavenging in the low-micromolar range. These findings highlight a synergy-driven mechanism integrating catalytic redox modulation, biomolecular interactions, and enhanced physicochemical compatibility.

## Abstract 2

Biomedical potential of Au/Pt/Cu nanofluid.

## Graphical abstract

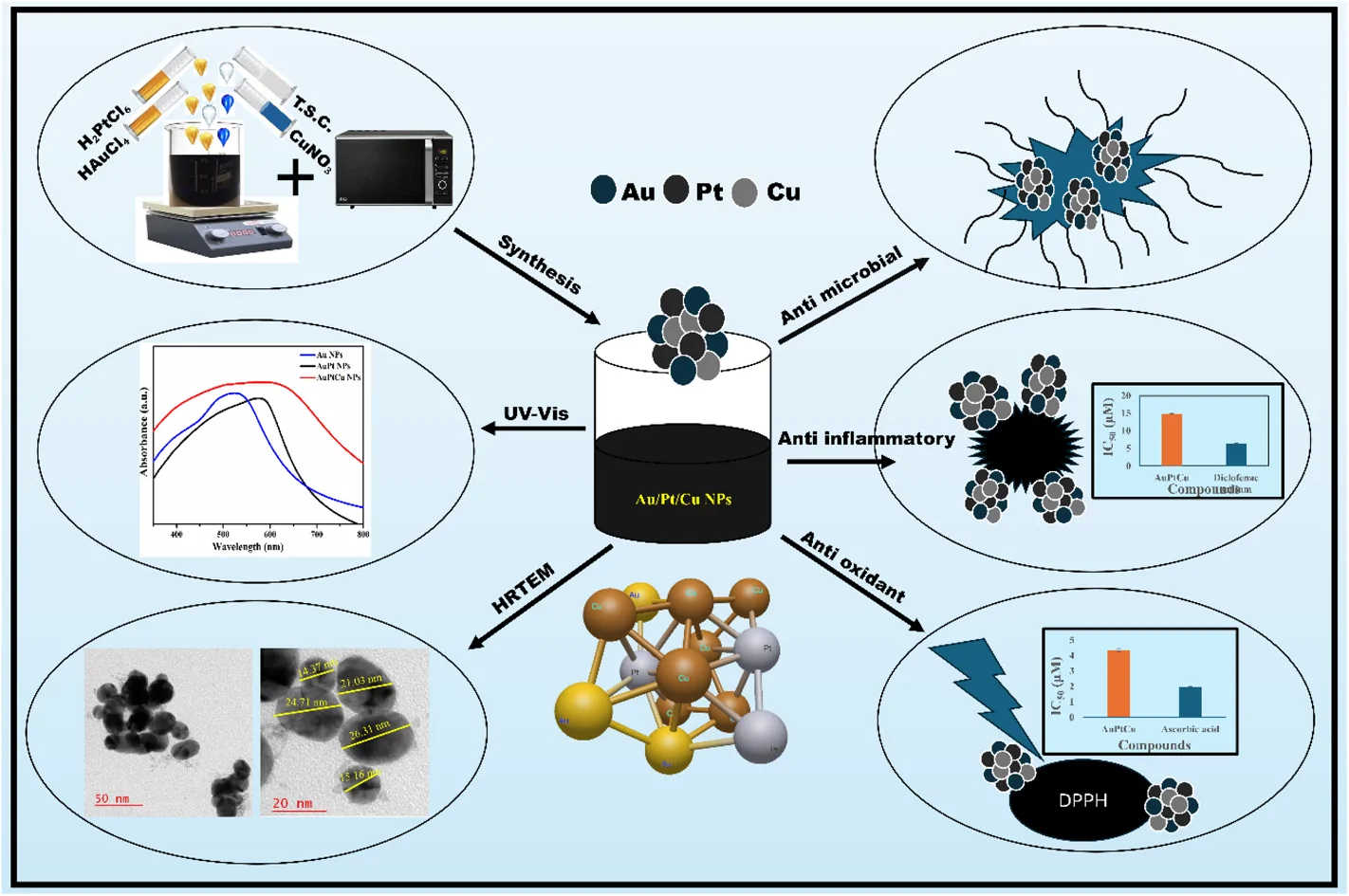

## 1 Introduction

Microbial diseases represent significant global health challenges and are caused by a variety of pathogenic microorganisms.^1,2^ Traditional treatments for microbial diseases often involve antibiotics or antiviral medications.^3,4^ However, the emergence of drug-resistant microbial strains and the limitations associated with conventional therapies have encouraged researchers to explore alternative therapeutic approaches. Inflammation is a serious issue for human beings, which is based on the damaged cells, pathogens, irritants *etc.* of the body. It is significant for the body's defense structure and has a crucial role in tissue homeostasis, repairing injuries and defending against infections.^5,6^ The inflammatory response is a precisely regulated and coordinated process involving various cells, signaling molecules, and vascular changes.^7^ The generation of many radicals and ions in the human body is the major reason behind the damage of lipids, fatty acids, DNA *etc.* which disturbs the biological balance of the body.^8^ These observed deformities are the major consequences of diabetes, liver failure, Parkinson, cancer *etc.*, which are death causing diseases. Hence there should be innovative substances which fulfill certain prerequisites to cure these illnesses, *viz.* antibacterial, anti-inflammatory and antioxidant vitality, fast action, minimal cytotoxicity, permitting clinically applicable methods of delivery *etc.*^9^ The utilization of metallic nanomaterials as biomedical agents has already shown to conform by most if not all the above-mentioned criteria.^10^

The metallic nanoparticles gain impressive attention due to a range of sizes and their morphologies.^11^ Metal-based nanoparticles have attracted significant attention due to their unique physicochemical properties, offering innovative opportunities in advanced applications such as catalysis,^12^ electronics,^13^ energy resources,^14^ energy storage,^15^ targeted drug delivery, bioimaging, biomedical, across medicinal, therapeutic,^16–18^ and diagnostic scopes.^19^

Antibacterial, anti-inflammatory, and antioxidant activities are among the biomedical applications of metal nanoparticles.^20–25^ The number of metallic components determines the classification of metal nanoparticles, which include monometallic, bimetallic, trimetallic, and so forth.^26–29^ When metallic nanoparticles are combined, they typically show new or improved properties that are not present in their constituent parts.^30–34^ Trimetallic nanoparticles are the focus of modern study because of this.^35–37^ Because of their superior catalytic efficacy when combined with single or bimetal, trimetallic nanoparticles are significant.^38–40^ In recent decades, the fabrication of multimetallic core–shell or alloy nanoparticles has drawn a lot of interest from both a fundamental and practical aspect.^38,41–43^ Current research trends are thus shifting from monometallic to multimetallic NPs because antibacterial activity against a certain bacteria became more with multimetallic components; trimetallic species were reactive against all the bacterial strains tried, as compared to their counterpart mono and bimetallic. For instance, mixed metal oxide CuO–NiO–ZnO nanoparticles demonstrated strong antibacterial properties and destructive effects on strains of *E. coli* and *S. aureus* bacteria; the NPs bonded to the bacterial cell wall and destroyed the cells.^44,45^ The green-synthesized trimetallic Au–Pt–Ag NPs showed promising results for antibacterial and anti-biofilm properties against *Candida*, *S. aureus*, *E. coli*, *E. faecium*, and *E. faecalis*.^43^ Alzahrani tested the antibacterial activities of the CuZnFe oxide NP against Gram negative *E. coli* and Gram-positive *E. faecalis*.^46^ Hussein *et al.* synthesized trimetallic nanocomposite (Ru/Ag/Pd)-Np and examined against *A. flavus*, *A. niger*, *C. albicans*, *C. glabrata*, *E. coli*, and *B.cereus*.^47^ Several trimetallic nanomaterials including Cu/Au/Pt,^48^ CuAuPd,^49^ PtCuCo,^50^ Au@AgPt,^51^ PdCuAu,^52^ AuAgPd, and FePt–Au^53^ with peroxidase-like strength have been synthesized and employed as catalyst for mercury ion detection, H_2_O_2_, glucose, or cancer cell.

In this study, Au/Pt/Cu trimetallic nanofluid (TMNF) (*i.e.*, a suspension of nanoparticles) was synthesized *via* a microwave (MW)-assisted chemical reduction method and its antibacterial activity was evaluated using serial dilution method which evaluates MIC value of the compounds. It has been already reported that Pt NPs have been successfully utilizing as antimicrobial source to restrict the develop of various bacterial microorganisms.^54^ Chemically stable and biocompatible Au nanoparticles exhibit a few distinctive properties as efficient antibacterial sources.^55^ Cu NPs can easily enter through the cell membrane and diffuse to the enzymatic action of the cell because of its excellent antimicrobial activity.^56–58^ Our goal was to combine the advantages and functions of three different metals while anticipating that each would have disadvantages that were countered by the other elements. So, the antimicrobial activity of the synthesized compound is worth mentioning in the present research. To elucidate the underlying mechanisms governing the observed biological activities, the experimental investigations were further supplemented by density functional theory (DFT) calculations. This integrated approach enables a detailed understanding of the electronic structure and reactivity of the Au–Pt–Cu nanofluid at the molecular level. In particular, frontier molecular orbital analysis and derived global reactivity descriptors including chemical hardness, softness, chemical potential, electronegativity, and electrophilicity provide critical insight into charge-transfer characteristics and interaction susceptibility with biological targets. Such correlations are essential for rationalizing the antimicrobial, anti-inflammatory, and antioxidant performance of the Au–Pt–Cu nanofluid and for establishing a strong structure–activity relationship (SAR).^59^

To ascertain a significant drug for the inflammation disorder, the inflammation inhibition property of the nanofluid was examined by BSA assay. We also evaluate the antioxidant property of the compound which compared with standard drug (ascorbic acid).

## 2 Experimental section

### 2.1 Materials

Hydrogen tetrachloroaurate(iii) trihydrate (HAuCl_4_·3H_2_O) and hexachloro platinic(iv) acid hydrate (H_2_PtCl_6_·*x*H_2_O) were procured from Hi-media Laboratories Pvt. Ltd, Mumbai. Copper nitrate (Cu(NO_3_)_2_) and trisodium citrate (Na_3_C_6_H_5_O_7_) were obtained from Merck, India. All the utilized chemicals were reagent-grade quality and used without further purification.

### 2.2 Synthesis of the Au/Pt/Cu trimetallic nanofluid

Employing a one-pot microwave assisted chemical route reduction method, Au/Pt/Cu nanofluid was synthesized. In short, an aqueous solution of HAuCl_4_·3H_2_O (0.1 wt%, 10 ml) was boil-heated while being gently stirred at the same time. Then dropwise added aqueous solution of trisodium citrate (0.1 wt%, 2 ml) into the solution vigorously stirring for approximately two minutes. The formation of bright magenta color of the solution from pale yellow confirmed stable gold (Au) nanoparticles nanofluid.^60,61^ In the Au nanofluid, aqueous solution of H_2_PtCl_6_·*x*H2O (0.1 wt%, 10 ml) was added along with aqueous solution of trisodium citrate (0.1 wt%, 2 ml), and the mixture was stirred simultaneously for four minutes. Then the solution was introduced to a microwave oven (LG – MC8084AB, Input 230 V, Input power Frequency 50 Hz, Microwave frequency 2450 MHz) operating at 360 Watts and heated for 3 minutes in a cyclic mode (20 s ON, 10 s OFF) to obtain the Au/Pt nanofluid.^62,63^ Finally, the trimetallic Au/Pt/Cu nanofluid was obtained by adding an aqueous CuNO_3_ solution (0.1 wt%, 10 ml) into the Au/Pt nanofluid with continuous stirring. The final solution was heated into a microwave oven in a cyclic mode (20 s ON, 10 s OFF) till 4 min and allowed to cool at room temperature. Natural cooling brought the colloidal dispersion of Au/Pt/Cu trimetallic nanofluid to ambient conditions.^64^ The whole synthesis procedure has been presented schematically in Fig. 1.

**Fig. 1:**
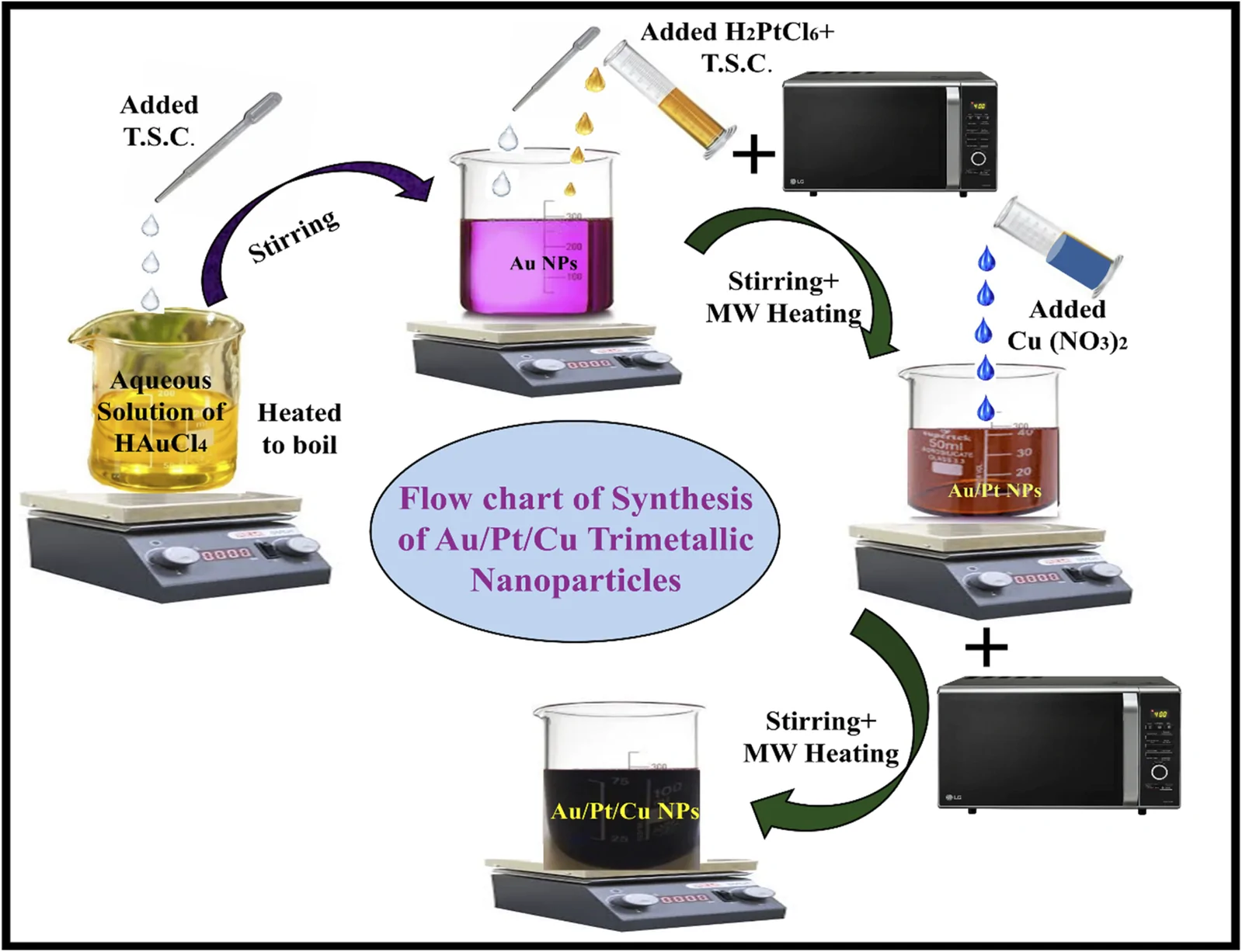
Schematic flow chart for the synthesis procedure of Au/Pt/Cu nanofluids.

### 2.3 Characterization

Microwave treatment of the nanofluids during synthesis was carried out using an LG MC8084AB microwave-convection oven, which operated at a supply voltage of 230 V AC at 50 Hz. This oven produced microwave radiation at a frequency of 2.45 GHz (2450 MHz), with a maximum output power of 900 W with five selectable power levels. Phase formation and crystallinity of the synthesized materials were examined by X-ray diffraction technique (XRD) (Proto A-XRD equipped with Cu K radiation = 1.5406). The surface chemistry and elemental composition of the solutions were investigated using a multipurpose X-ray photoelectron spectrometer (XPS). The chemical solution was poured onto the doped silicon substrates using the drop cast method and left to dry overnight in the fume hood. XPS measurements of prepared Au/Pt/Cu sample were carried out using an Axis Supra XPS system (Kratos Analytical) equipped with a monochromatic Al-Kα X-ray source (1486.7 eV) and a large area slot mode detector (approximately 300 µm × 800 µm analysis areas). Microstructural characterization at high magnifications and reciprocal space analysis was carried out using a high-resolution transmission electron microscope (HR-TEM: FEI Tecnai G2 F30 STWIN) at 300 keV. The surface morphology of prepared trimetallic Au/Pt/Cu nanoparticles was examined by a field emission scanning electron microscope (FESEM; Zeiss Gemini SEM, Carl Zeiss). For the elemental analysis of the trimetallic nano-species, Energy Dispersive X-ray Spectroscopy (EDS) and elemental mapping were carried out by using same FESEM (Zeiss Gemini SEM, Carl Zeiss) equipped with a SwiftED 3000 SDD detector. Optical characterization was carried out using UV-vis spectrometer (Perkin Elmer Lambda-35 double beam spectrophotometer). FTIR analysis was performed to detect the formed functional groups by using Perkin Elemer FTIR spectrometer in the range of 500 and 4000 cm^−1^. All the experiments were executed duplicate to establish the reproducibility. Hydrodynamic size and zeta potential of the aqueous Au, Au/Pt, and Au/Pt/Cu nanoparticles were measured using a Zetasizer Nano ZS (Malvern Instruments, UK) with water as the dispersant. Measurements were performed at 25 °C. For hydrodynamic-size analysis, samples were measured in disposable sizing cuvettes, whereas zeta-potential measurements were performed using clear disposable zeta cells. According to the new instrument records, the zeta-potential analysis comprised 14 runs for the Au sample and 12 runs each for the Au/Pt and Au/Pt/Cu samples. The corresponding size-distribution and apparent zeta-potential profiles were used for comparison among the three nanoparticle systems.

### 2.4 Biomedical portion activities

#### 2.4.1 Antimicrobial activity

The antimicrobial activity of the Au/Pt/Cu nanofluids dispersion was evaluated by serial broth dilution against *Pseudomonas aeruginosa* (MTCC 424), *Staphylococcus aureus* (MTCC 2901), *Escherichia coli* (MTCC 732), *Bacillus subtilis* (NCIM 2063), *Rhizopus oryzae* (MTCC 262), and *Candida albicans* (MTCC 227). Measurements were performed in triplicate. Ciprofloxacin and fluconazole served as positive antibacterial and antifungal controls, respectively, and dimethyl sulfoxide (DMSO) served as the vehicle/negative control.^59^

A stock dispersion (100 µg mL^−1^) was prepared in DMSO and diluted with sterile nutrient broth or potato dextrose broth to obtain concentrations of 3.12–50 µg mL^−1^. Standardized microbial inocula from fresh cultures were added aseptically. Bacterial cultures were incubated at 37 °C for 24 h, and fungal cultures were incubated at 28 °C for 48 h.^60^

The MIC was defined as the lowest tested concentration showing no visible growth relative to the corresponding control. Because the sample is a multicomponent particulate ensemble and does not possess a unique molecular mass, nanoparticle doses are reported primarily in µg mL^−1^ rather than as molar concentrations. This avoids an assumption of exact stoichiometry and permits direct traceability to the experimental dilution series.

#### 2.4.2 Anti-inflammatory activity

The ability of the sample to inhibit heat-induced BSA denaturation was evaluated in triplicate and compared with diclofenac sodium. Sample concentrations of 12.5–200 µg mL^−1^ were prepared in DMSO and diluted with phosphate buffer (0.2 M, pH 7.4). Four millilitres of each sample solution were mixed with 1 mL of BSA solution (1 mM in phosphate buffer).^24,31,68^ The mixtures were incubated at 37 °C for 20 min and then heated at 70 °C for 15 min. After cooling to room temperature, absorbance was measured at 660 nm. Percentage inhibition of protein denaturation was calculated relative to the vehicle control.
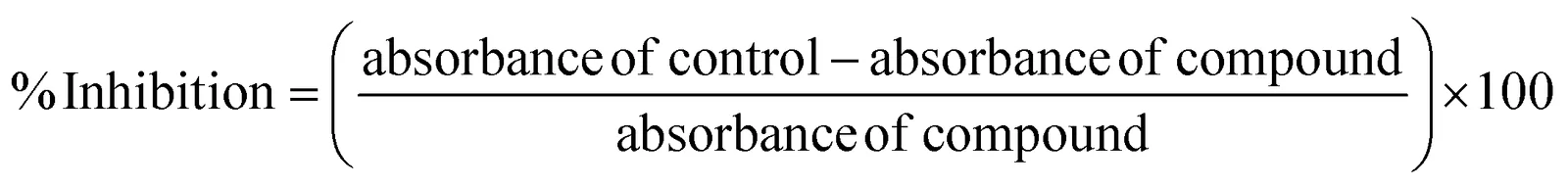


Apparent IC_50_ values were estimated from the concentration-response data.

#### 2.4.3 Antioxidant activity

DPPH radical-scavenging activity was evaluated in triplicate. Sample solutions (12.5, 25, 50, 100, and 200 µg mL^−1^) were prepared in DMSO, and ascorbic acid was used as the reference antioxidant. One millilitre of each sample solution was mixed with 1 mL of DPPH solution prepared by dissolving 1 mg of DPPH in 20 mL of DMSO.^68–70^ The mixtures were shaken and incubated in the dark for 30 min. Absorbance was recorded at 517 nm against the appropriate blank, and percentage radical scavenging was calculated relative to the DPPH control.
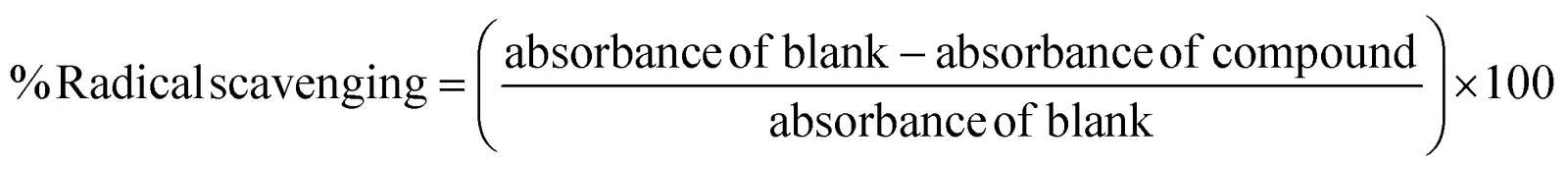


The results obtained were used to calculate the IC_50_ value which was further compared with ascorbic acid.

### 2.5 Computational methodology

Quantum-chemical calculations were performed with ORCA 6.1.1. Structures were optimized at the PBE0 DFT level.^71^ Scalar relativistic effects were treated using the zeroth-order regular approximation (ZORA). SARC-ZORA-TZVP basis sets were applied to Au and Pt, and ZORA-def2-TZVP was used for Cu. Coulomb and exchange integrals were evaluated with the RIJCOSX approximation and automatically generated auxiliary basis sets. Dispersion was included using D3BJ. Analytical frequency calculations were used to verify stationary points and obtain quasi-RRHO thermochemical corrections at 298.15 K. Dipole moments, quadrupole moments, and polarizabilities were calculated including picture-change effects. The small cluster model was selected for computational tractability and is interpreted as a qualitative representation of local Au–Pt–Cu electronic interactions.

## 3 Results and discussions

### 3.1 Phase and composition analysis

The crystalline structure and phase composition of synthesized trimetallic Au/Pt/Cu nanofluid was investigated by X-ray diffraction (XRD). The XRD pattern recorded over 2*θ* = 25–80° contains reflections consistent with face-centred-cubic Au, Pt, and Cu phases shown in Fig. 2. The diffraction pattern exhibits well defined peaks, which aligned with standard JCPDF patterns of Au, Pt and Cu. The diffraction peaks obtained at 38.45°, 45.50°, 64.15°, and 77.83° corresponds to standard (111), (200), (220) and (222) plane of Au, reflecting the presence of face-centered cubic (fcc) structure of gold (JCPDF file 04-0784).^65^ It is well known that the diffraction peaks of Pt are very close to those of Au when compared to the corresponding standard JCPDF file (JCPDS No. 04-0802). The diffraction peak observed at 39.15° and 45.65° are related to Pt (111), Pt (200) respectively, and exhibit slightly overlap with the corresponding peaks of Au. However, the peak observed at 67.27° is well distinct and related to standard Pt (220) plane.^66–68^ It is very hard to identify the peaks of Au and Pt, when both metals are present. In the XRD pattern, the peaks obtained at 43.45°, 50.10°, and 75.32° correspond to Cu (111), Cu (200) and Cu (220) planes, respectively and are in well-matched agreement with standard JCPDF of Cu (JCPDF 04-0836). Moreover, slight shifts in the peak positions compared to standard JCPDS of Au, Pt and Cu suggest lattice distortion, resulting from the formation of alloy and atomic substitution among Au, Cu, and Pt atoms. Three additional diffraction peaks observed at 27.43°, 31.75° and 56.60° correspond to intermetallic Au3Cu (100), CuAu (031) and Au3Cu (200) (JCPDF 27-0156 & 34-1302).^35,38,64^ The appearance of these additional peaks indicates the strong atomic interactions between Au and Cu during synthesis procedure and resulting in the formation of an Au3Cu and CuAu alloy.

**Fig. 2:**
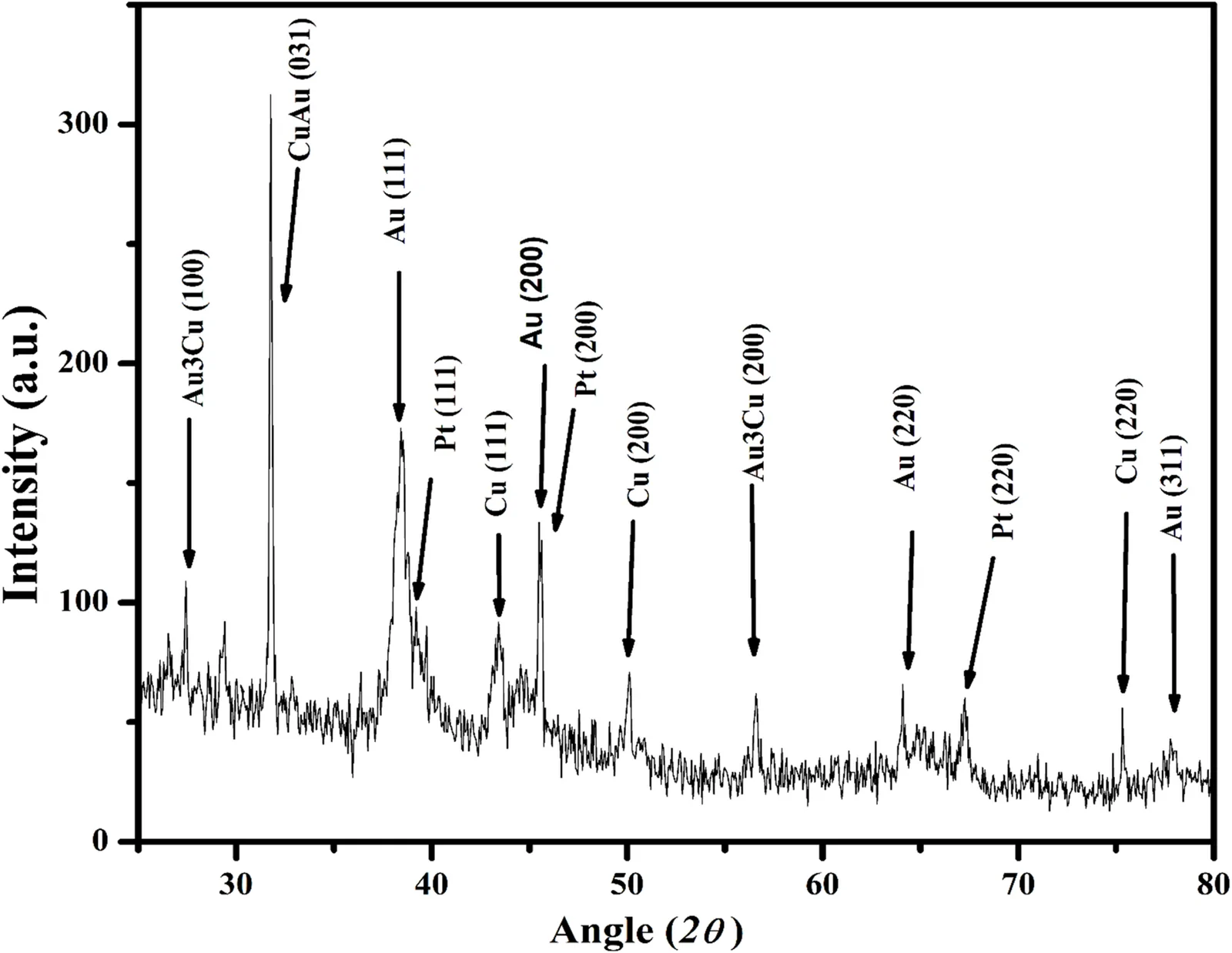
XRD pattern of trimetallic Au/Pt/Cu nanofluid.

Overall, the XRD result of trimetallic Au/Pt/Cu indicates that nanofluid consists of Au/Pt random alloy combined with Cu nanoparticles. These types of lattice modifications are usually observed in multimetallic nanoparticles and significantly impact their structural and catalytic potential.

The XPS measurements have been used to study the chemical structure of Au/Pt/Cu nanofluid. Wide scan XPS survey of the nanofluid reveals presence of C, O, Au, Pt and Cu respectively. Fig. 3(a) shows its core level spectra deconvoluted with best fitting parameters based on casa xps software. Fig. 3(b) clearly depicts core level spectra of C 1s deconvoluted into 4 peaks with binding energies 281.9, 283.3, 285.4, and 289.9 eV which correspond to C=C, C–O, C=O and O–C=O bonds respectively.^69^Fig. 3(c) exhibits core level spectra of O 1s deconvoluted into two components located at ∼530.4 and ∼531.8 eV, which are attributed to lattice oxygen and surface hydroxyl species, respectively. Fig. 3d shows the deconvoluted XPS spectrum of gold (Au), consisting of two symmetrical peaks. The spin–orbit splitting component for Au is 3.7 eV, and the peak located at 83.9 eV reflecting the presence of Au 4f_7/2,_ while another peak centred at 87.5 eV indicating 4f_5/2_, which could be attributed to Au atoms with a chemical valence state of zero. The fitted XPS spectrum of Pt 4f, consisting of two distinct peaks and is presented in Fig. 3e. The peak at 71.7 eV indicates the presence of metallic Pt 4f and corresponds to Pt 4f_7/2_. The spin–orbit splitting value for Pt 4f is 3.35 eV, whereas the other peak centred at 73.4 eV represents metallic Pt 4f_5/2_.^66^ The high resolution XPS spectrum of Cu 2p has shown in Fig. 3(f). As shown in Fig. 3(f), the fitted XPS spectrum of Cu 2p exhibited a two-band structure and a perfect doublet, indicating a spin–orbit splitting value of 19.75 eV for the Cu 2p. The fitted XPS peaks of Cu 2p are symmetrical and located at 930.1 and 949.75 eV, corresponding to the Cu 2p_3/2_ Cu (0) and Cu 2p_1/2_ peaks Cu(ii), reflecting the existence of Cu in samples. On the other hand, Cu 2p showed the weak satellite peaks observed at 940.9 & 960.1 eV are related to Cu 2p_3/2_ and Cu 2p_1/2_ transitions with perfect spin-obit splitting of 19.75 eV. Overall, the XPS spectrum exhibits the presence of Cu 2p in the nanofluid.^64^

**Fig. 3:**
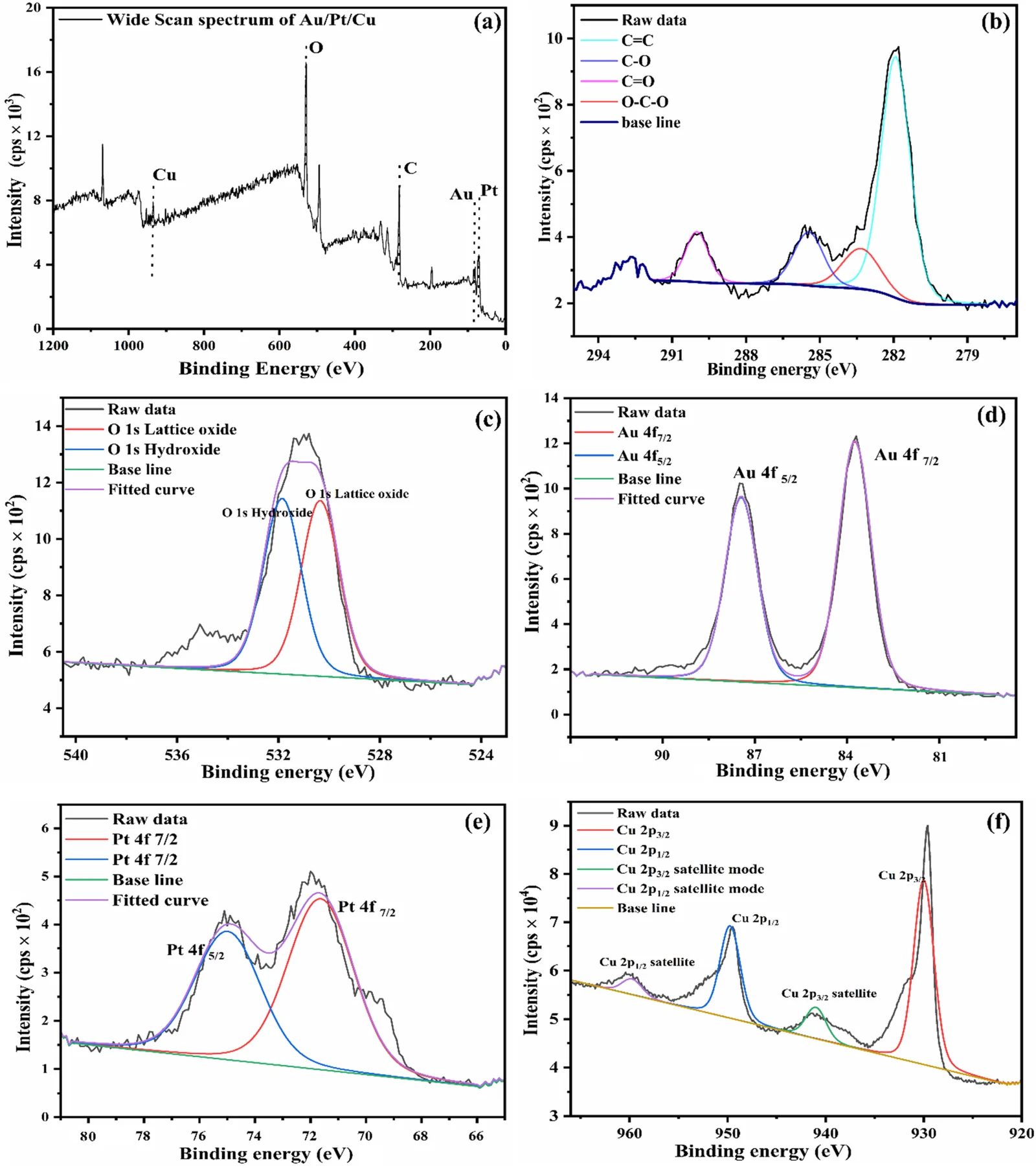
Deconvoluted XPS images of Au/Pt/Cu nanofluid (a) wide scan spectrum of Au/Pt/Cu, (b) fitted spectrum of C 1s, (c) fitted spectrum of O 1s, (d) fitted spectrum of Au 4f, (e) fitted spectrum of Pt 4f, (f) fitted spectrum of Cu 2p.

### 3.2 Microstructural analysis

Multimetallic nanoparticles are usually synthesized *via* chemical route reduction method by successive reduction of respective precursors. Usually in the process of successive reduction of the metal salts leads to form a core/shell, composite, or alloy type NPs.^41,64^ The microstructure of the synthesized trimetallic Au/Pt/Cu nanofluid was analyzed using transmission electron microscopy (TEM) and shown in Fig. 4. Initially, TEM micrographs were used to examine the diameters of the nanoparticles as shown in Fig. 4a and b. Fig. 4(a) clearly depicts that most of the trimetallic Au/Pt/Cu nanoparticles are uniform, spherical, and well dispersed. Fig. 4(b) depicts that Au/Pt/Cu had a particle size distribution between 16 and 26 nm at a magnified scale bar of 20 nm. Based on randomly selected nanoparticles in Fig. 4(a), the corresponding size-distribution histograms plot of nanoparticles indicates that most of the nanoparticles appear in the range of 16–26 nm, and it is displayed in the adjacent Fig. 4(c).

**Fig. 4:**
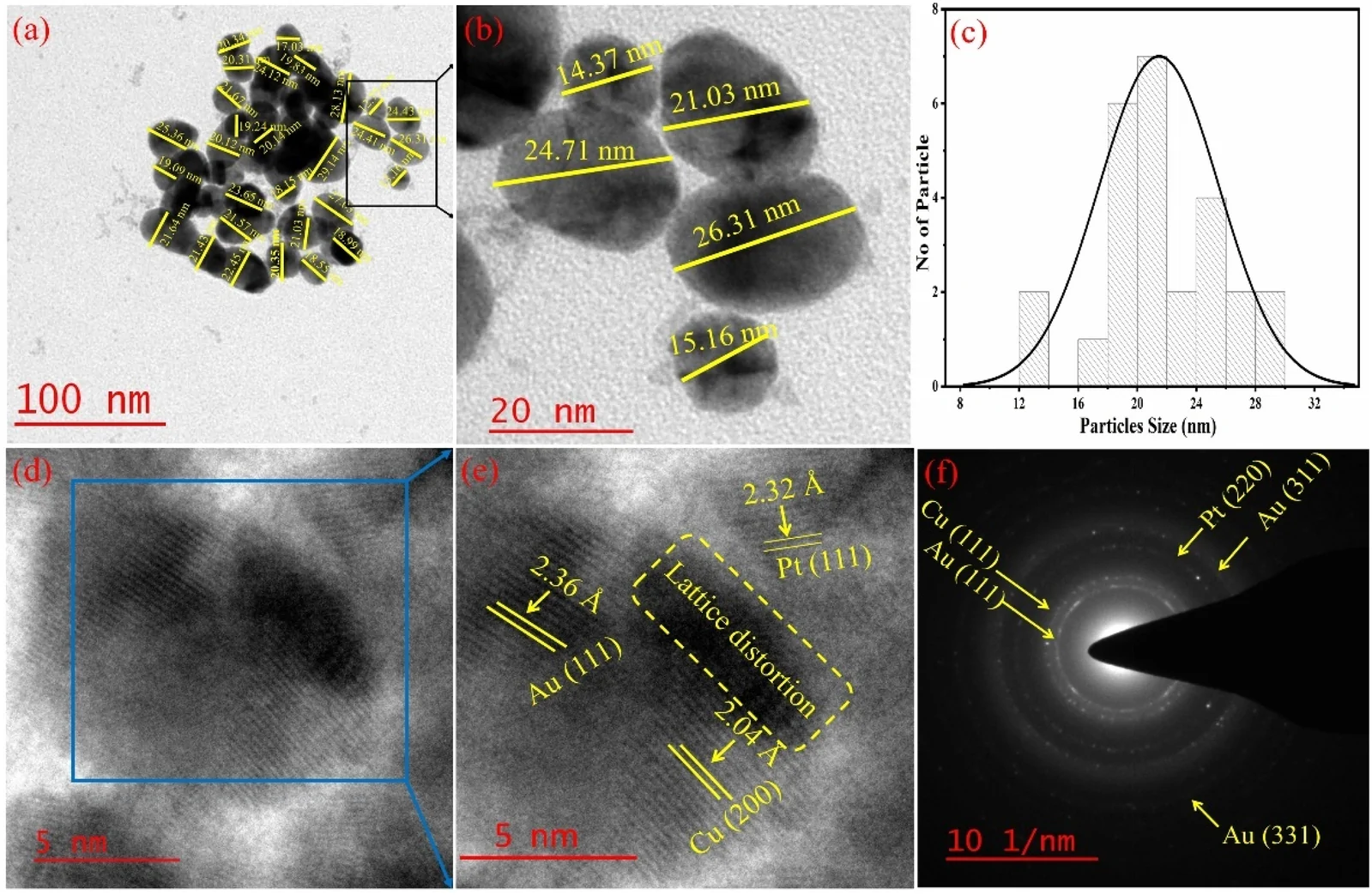
TEM images of (a and b) Au/Pt/Cu nanofluid at 100 nm and 20 nm resolution (c) Particle size distribution histogram of Au/Pt/Cu nanofluids (d and e) HRTEM images of Au/Pt/Cu nanofluids (f) SAED pattern.

High resolution transmission electron microscopy (HRTEM) image of the nanofluids is shown in Fig. 4(d) at the scale of 5 nm. The smaller size could be the consequence of the mixed nanostructures combining to produce a larger particle. It suggests alloying of particles, as the HRTEM pictures of the lattice fringe make clear. The magnified view of the selected area from Fig. 4(d) is shown in Fig. 4(e), which depicts the lattice fringes of the Au, Pt and Cu. Fig. 4(e) clearly demonstrates the lattice spacings of 2.36 Å, 2.32 Å and 2.04 Å for Au (111), Pt (111), and Cu (200) respectively, along with a lattice distortion. The trimetallic properties of the as-formed particles are demonstrated by this image, along with the simultaneous presence of distinct metallic alloy zones constructed up of the three metals Au, Pt, and Cu. The SAED pattern of the trimetallic nanofluid is shown in Fig. 4(f), demonstrates the bright circular rings with concentrical spots, reflecting the polycrystalline nature of the Au/Pt/Cu NPs. The observed *d*-spacing of the circular rings are 2.334, 2.076, 1.942, 1.232 and 0.942 Å correspond well to the Au (111), Cu (111), Pt (111), Au (311) and Au (331) planes respectively, confirming the face-cantered cubic (FCC) structure of the nanoparticles.^43^ Overall, it is perfectly indisputable that the particle is trimetallic.

EDS confirms the presence of Au, Pt, and Cu in the analysed region. The inset in Fig. 5(a) reports weight percentages of 24.5% Au, 21.7% Pt, and 53.8% Cu and atomic percentages of 11.5% Au, 10.3% Pt, and 78.3% Cu. These correspond to normalized Au : Pt : Cu ratios of approximately 1.13 : 1 : 2.48 by weight and 1.12 : 1 : 7.60 by atomic percentage. Fig. 5(b) provides the corresponding SEM micrograph of Au/Pt/Cu trimetallic nanofluid showing spherical molecules. The elemental mapping technique further provided a compositional analysis that was more visually engaging. The distribution ranges of Au (Fig. 5(c)), Pt (Fig. 5(d)), and Cu (Fig. 5(e)) appear to be overlapping with Cu becoming the most abundant of the trio. Overall distribution of the three metals was almost uniform across the entire region of investigation.

**Fig. 5:**
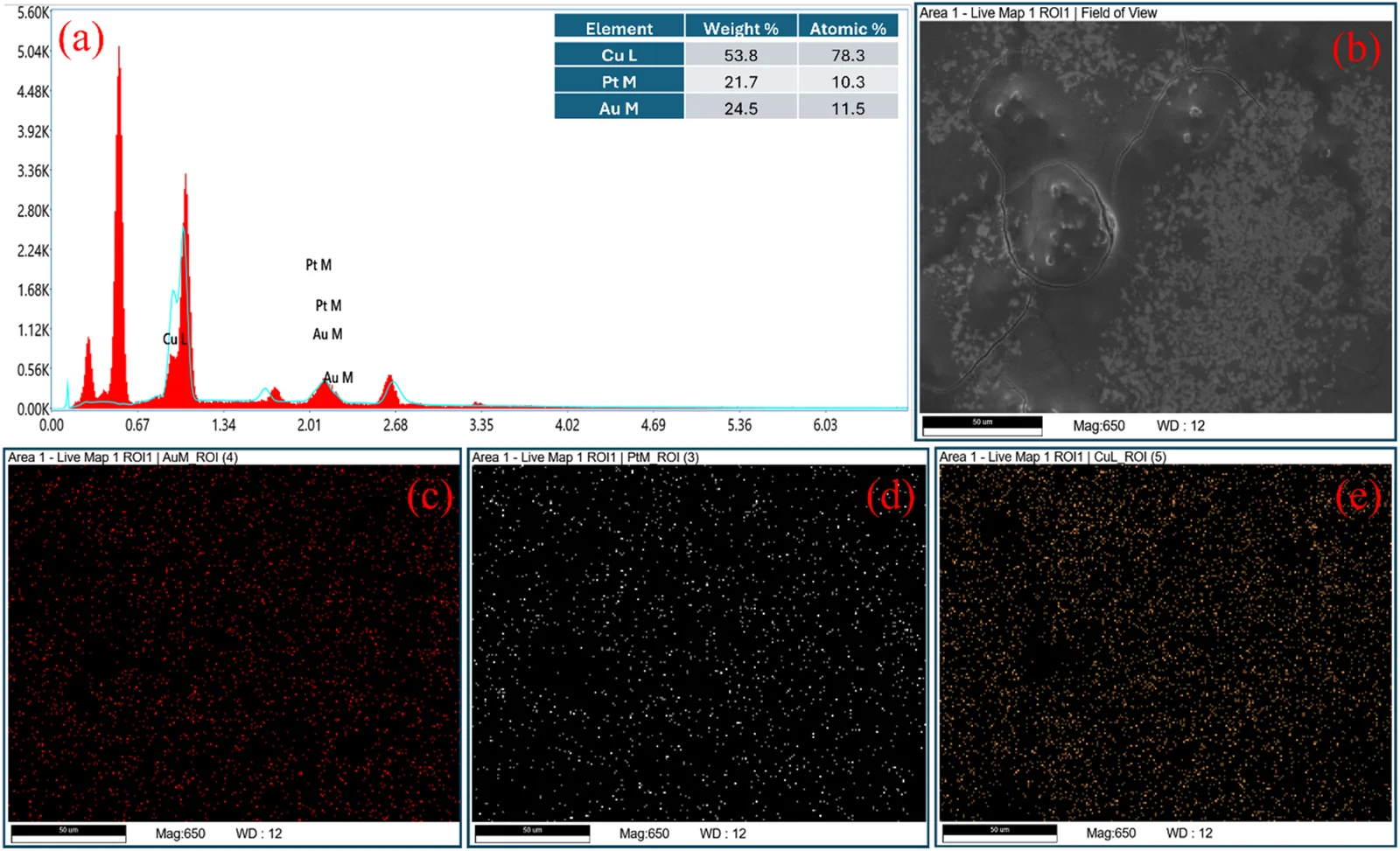
EDX, SEM and mapping of trimetallic Au/Pt/Cu nanofluid.

### 3.3 Optical analysis

Surface plasmon resonance (SPR) is the phenomenon of oscillation when frequency of conduction electrons of metal nanoparticles resonates with the incident radiation. This interaction is affected by the size and shape of the nanoparticles, the distance between them, and the dielectric environment of the metal nanoparticles. The monometallic Au nanofluid in this investigation shows SPR peaks with a 525 nm centre.^65^ The typical surface plasmon resonance caused by spherical gold nanoparticles is typically responsible for the peak at 525 nm. The asymmetrical irregular shape of gold nanoparticles was not detected by UV-vis microscopy investigation depicted in Fig. 6(a). The Au/Pt bimetallic nanoparticles show a similar absorption band at 575 nm. This absorption band attributed red shift with higher absorption band. This shifting further authenticates the deposition of Pt layer on the Au nanofluid surface; and not typically by core–shell structure of Au/Pt nanofluid.^42^ In the case of core–shell formation, Pt would have covered the Au core, either completely or partially suppressing the Au SPR bands completely or partially. The TEM micrograph (Fig. 4), which clearly shows that the Au/Pt bimetallic nanoparticles tend to approach each other closer or more densely than the monometallic Au, contradicts the conclusion that the incorporation of Pt may have decreased the concentration of agglomeration. The lower energy band was blue shifted to the center ∼614 nm; it became almost imperceptibly weak. The Au/Pt/Cu trimetallic exhibits a broad absorption band at *l* > 510 nm (Fig. 6a). The SPR of Pt was humified probably by AuCu, giving rise to continuous absorption band. It has been observed that Cu nanoparticles possess SPR band within the range of 550–570 nm.^70^ In the case of alloy formation with the incorporation of Cu into the trimetallic Au/Pt/Cu, the surface plasmon of Au was red shifted from 510 nm to 590 nm and broadened. It is projected that the Au SPR band would redshift, resulting in a broadening of the peak.

**Fig. 6:**
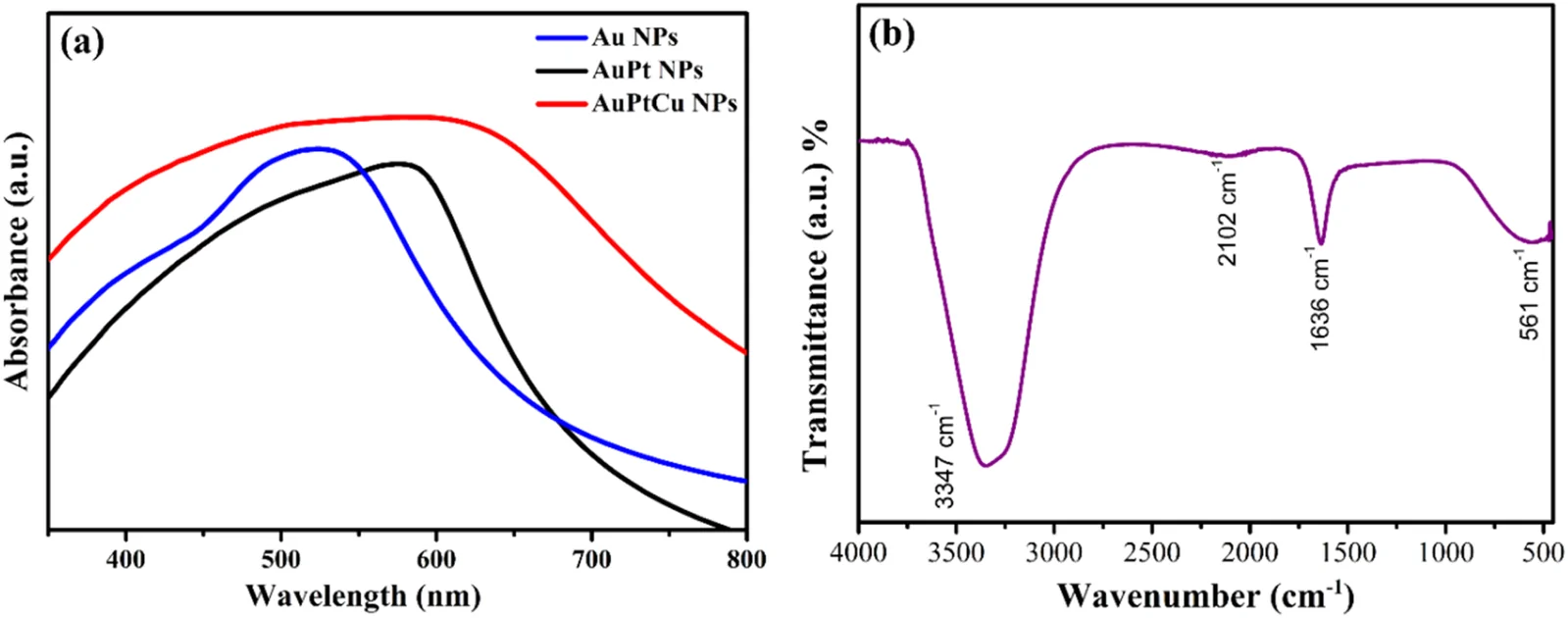
(a) UV-vis absorption and (b) FTIR spectra of the trimetallic Au/Pt/Cu nanofluid.

The FTIR spectrum (Fig. 6b) contains a broad O–H band near 3347 cm^−1^ and a feature near 1636 cm^−1^ that may include contributions from adsorbed water and carboxylate-containing citrate-derived surface species. A low-wavenumber band in the 520–570 cm^−1^ region may contain metal–oxygen contributions and is consistent with the possibility of partial surface oxidation, particularly for Cu.

Dynamic light scattering (DLS) and zeta-potential measurements were used to evaluate the hydrodynamic size distribution and surface charge of the Au, Au/Pt, and Au/Pt/Cu nanofluids (Fig. 7). The Au NPs exhibited a *Z*-average hydrodynamic diameter of 16.96 nm with a polydispersity index (PDI) of 0.306. Following Pt incorporation, the *Z*-average increased to 33.33 nm for the Au/Pt NPs, with a PDI of 0.670. Subsequent incorporation of Cu resulted in a *Z*-average diameter of 37.57 nm and a PDI of 0.712 for the Au/Pt/Cu NPs. The progressive increase in *Z*-average indicates a change in the hydrodynamic environment following sequential metal incorporation. Since DLS reports the hydrodynamic diameter, the measured value can include contributions from the nanoparticle surface layer and the surrounding electrical double layer, in addition to the inorganic core. The higher PDI values obtained for the Au/Pt and Au/Pt/Cu samples indicate broader hydrodynamic size distributions than that of the Au sample.^71,72^ The volume-based distributions were dominated by nanoscale populations. The major size populations were centred at 13.43 nm for Au NPs (100.0% by volume), 15.92 nm for Au/Pt NPs (99.6% by volume), and 15.44 nm for Au/Pt/Cu nanofluids (99.5% by volume). Minor larger-size populations were detected for the Au/Pt sample at approximately 490.3 nm (0.4%) and 4.69 µm (0.1%), whereas the Au/Pt/Cu sample showed minor populations at approximately 520.0 nm (0.4%) and 5.34 µm (0.1%). Thus, although the dominant volume fraction remained within the nanoscale range, the multi-metallic samples contained a small contribution from larger scattering species or aggregates, which is consistent with their higher *Z*-average and PDI values. Zeta-potential analysis showed that all three nanoparticle systems possessed negatively charged surfaces. The apparent zeta-potential distribution was centred at −43.9 mV for Au NPs, −45.0 mV for Au/Pt NPs, and −55.2 mV for Au/Pt/Cu nanofluids, with corresponding zeta deviations of 6.24, 6.18, and 6.21 mV, respectively. In each case, a single zeta-potential population accounted for 100% of the reported distribution. The increase in the magnitude of the negative zeta potential after sequential Pt and Cu incorporation indicates modification of the nanoparticle interfacial charge. The relatively large negative surface potentials are consistent with appreciable electrostatic repulsion between particles under the measurement conditions, with the Au/Pt/Cu sample exhibiting the greatest magnitude of negative surface charge.

**Fig. 7:**
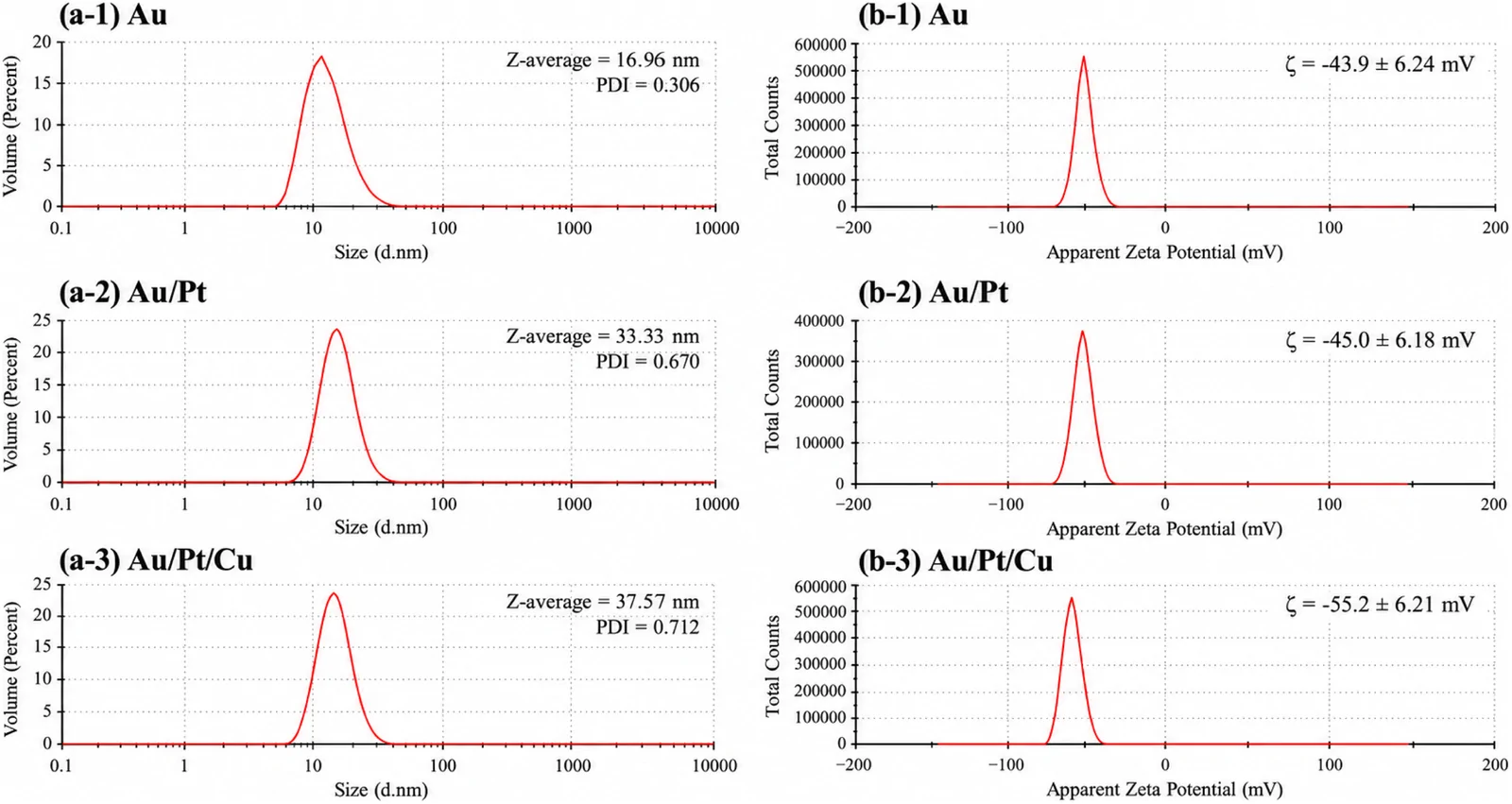
Dynamic light scattering (DLS) and zeta-potential characterization of Au, Au/Pt, and Au/Pt/Cu nanoparticles. (a-1–a-3) Hydrodynamic size distributions of Au, Au/Pt, and Au/Pt/Cu NPs, respectively; (b-1–b-3) corresponding apparent zeta-potential distributions. The *Z*-average hydrodynamic diameters were 16.96, 33.33, and 37.57 nm, whereas the apparent zeta potentials were −43.9, −45.0, and −55.2 mV for Au, Au/Pt, and Au/Pt/Cu NPs, respectively.

Overall, the DLS and zeta-potential results demonstrate that sequential incorporation of Pt and Cu altered both the hydrodynamic and electrokinetic properties of the nanoparticles. The Au sample displayed the narrowest hydrodynamic size distribution, whereas the Au/Pt and Au/Pt/Cu samples showed broader distributions together with strongly negative surface potentials. These findings support progressive modification of the nanoparticle interfacial properties across the Au, Au/Pt, and Au/Pt/Cu series.

### 3.4 Biomedical activity

Multi-metallic nanomaterials are increasingly explored in biomedical research because the combination of different metals within a single nanostructure often leads to cooperative physicochemical properties that cannot be achieved by individual components alone. The integration of multiple metal centres can modulate redox behaviour, catalytic activity, and surface chemistry, thereby influencing key biological processes such as microbial survival, protein stability, and free-radical reactions. In this study, the Au/Pt/Cu trimetallic nanofluid (TMNF) was investigated as a multifunctional platform capable of interacting with diverse biological targets. Each metal present in the system contributes a distinct functional role. Copper is well known for its redox activity and its ability to undergo reversible Cu^+^/Cu^2+^ cycling, which can promote the generation of reactive oxygen species (ROS) in biological environments. Platinum provides catalytically active surfaces that facilitate redox transformations involving peroxides and related species. Gold, on the other hand, exhibits strong affinity toward sulfur-containing and phosphorus-containing biomolecular groups, enabling interactions with proteins, membrane components, and nucleic acids. The collective presence of these metals therefore enables the nanofluid to interact with multiple cellular components simultaneously. In addition, partial charge redistribution between the metal centres and surrounding ligands enhances the lipophilic character of the nanostructure. Increased lipophilicity can improve interactions with lipid membranes and facilitate cellular uptake. Taken together, these attributes suggest that the Au/Pt/Cu nanofluid may exert biological activity through several complementary pathways, which is reflected in its antimicrobial, anti-inflammatory, and antioxidant behavior described below.

#### 3.4.1 Antimicrobial activity

##### 3.4.1.1 Mechanistic rationale

The antimicrobial activity of the trimetallic nanofluid is likely governed by a combination of physicochemical and biochemical interactions. One potential mechanism involves the generation of reactive oxygen species through metal-mediated redox reactions, which can induce oxidative stress within microbial cells. Elevated ROS levels are known to damage membrane lipids, proteins, and nucleic acids, ultimately impairing cellular viability. A second contributing factor may be the catalytic role of platinum, which can influence intracellular peroxide balance and disrupt the oxidative homeostasis necessary for microbial survival. In parallel, the strong affinity of gold for thiol and phosphate groups enables interactions with enzyme active sites and nucleic acid backbones, potentially interfering with essential metabolic pathways and genetic processes. Another important aspect is the enhanced lipophilicity of the trimetallic system. Increased hydrophobic interaction with biological membranes may facilitate penetration through microbial cell walls and membranes. The combined effects of oxidative damage, enzymatic inhibition, and membrane disruption can lead to increased membrane permeability, metabolic imbalance, and eventual growth inhibition of microbial cells.

##### 3.4.1.2 Experimental methodology

The MIC experiment was performed in nutrient broth for bacteria and potato dextrose broth for fungi, with three independent measurements for each condition. The test panel comprised *S. aureus*, *B. subtilis*, *E. coli*, *P. aeruginosa*, *R. oryzae*, and *C. albicans*. Ciprofloxacin and fluconazole were the positive controls, and DMSO was the vehicle/negative control.

##### 3.4.1.3 Antimicrobial performance

The trimetallic nanofluid exhibited consistent inhibitory activity against all tested bacterial strains, with an MIC value of 0.0137 µmol mL^−1^ for *S. aureus*, *B. subtilis*, *E. coli*, and *P. aeruginosa*. For the fungal organisms *R. oryzae* and *C. albicans*, the MIC value was 0.0068 µmol mL^−1^ (Table 1 and Fig. 8), indicating relatively stronger antifungal activity. Thus, the compound confirmed the highest inhibition efficacy against fungal strains.^73,74^ When compared with the reference drugs, ciprofloxacin displayed greater antibacterial potency, with MIC values of 0.0047 µmol mL^−1^, which is consistent with its established clinical effectiveness. Nevertheless, the trimetallic nanofluid demonstrated uniform inhibitory activity across both Gram-positive and Gram-negative bacterial species. Interestingly, against *C. albicans*, the nanofluid showed stronger activity than fluconazole under the same experimental conditions (0.0068 *vs.* 0.0102 µmol mL^−1^). However, fluconazole remained slightly more effective against *Rhizopus oryzae* (0.0051 µmol mL^−1^).

**Table 1:** Antimicrobial activity of synthesized Au/Pt/Cu nanofluid

Compound	Gram +ve bacteria		Gram −ve bacteria		Fungi	
	*S. aureus*	*B. subtilis*	*E. coli*	*P. aeruginosa*	*R. oryzae*	*C. albicans*
Au/Pt/Cu	0.0137	0.0137	0.0137	0.0137	0.0068	0.0068
Ciprofloxacin	0.0047	0.0047	0.0047	0.0047	—	—
Fluconazole	—	—	—	—	0.0051	0.0102

**Fig. 8:**
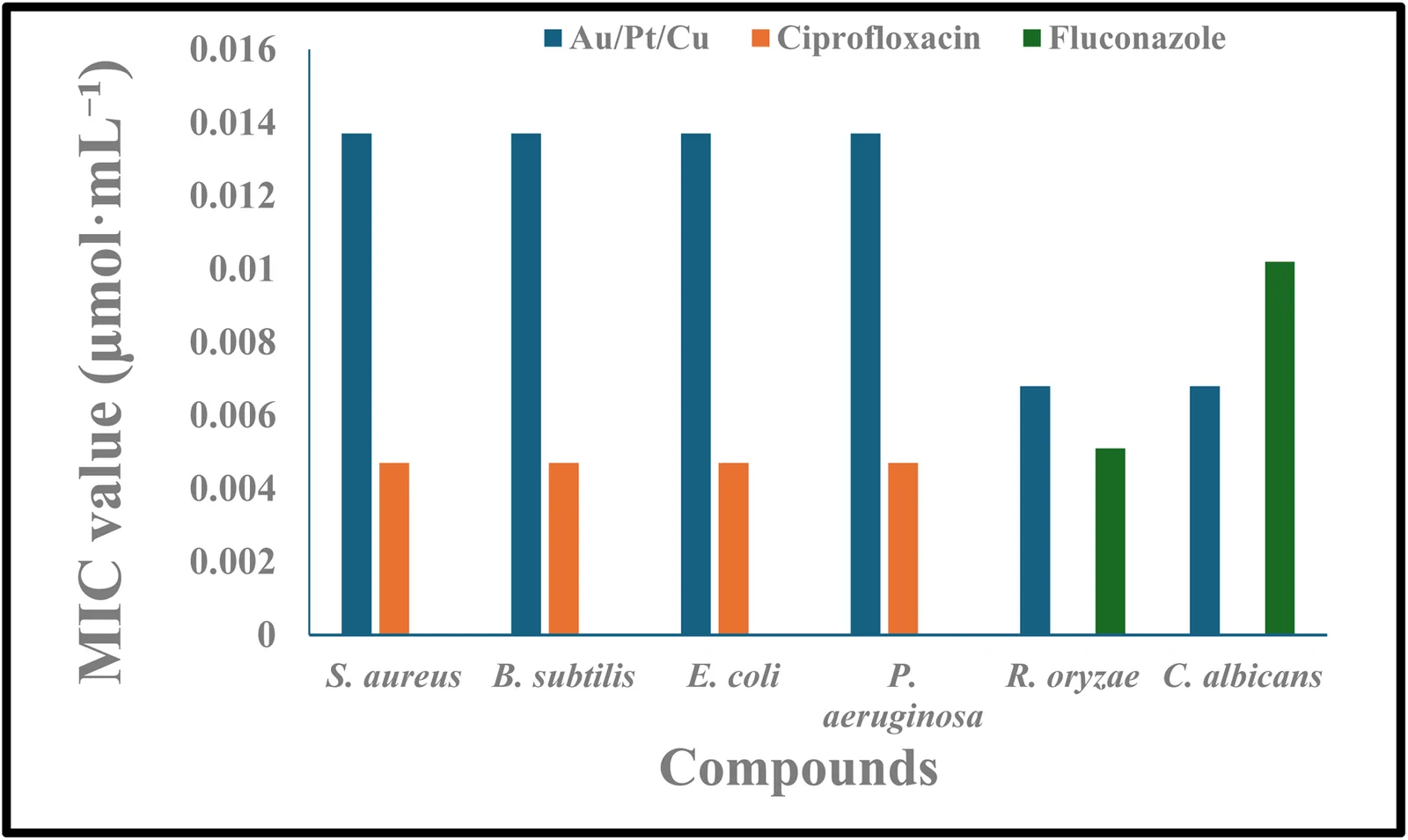
Antimicrobial activity of the Au/Pt/Cu (MIC values).

##### 3.4.1.4 Implications and future studies

The results suggest that the Au/Pt/Cu nanofluid possesses broad-spectrum antimicrobial potential with notable antifungal effectiveness. Such dual activity is particularly valuable in polymicrobial infections, where both bacterial and fungal pathogens may coexist, as is commonly observed in chronic wounds or infections associated with medical devices. To further understand the antimicrobial properties of this system, additional studies should examine bactericidal activity by determining minimum bactericidal concentrations (MBC). Time-kill assays, biofilm inhibition studies, and post-antibiotic effect analyses would also provide deeper insights into its therapeutic potential. Moreover, evaluating possible synergistic interactions with conventional antibiotics may help identify combination strategies for enhanced antimicrobial therapy.

#### 3.4.2 Anti-inflammatory activity

##### 3.4.2.1 Principle of the assay

The anti-inflammatory potential of the trimetallic nanofluid was examined using the heat-induced bovine serum albumin (BSA) denaturation assay. Protein denaturation is commonly associated with inflammatory processes because structural destabilization can lead to aggregation and activation of inflammatory signaling pathways. Therefore, compounds capable of protecting proteins from thermal denaturation may exhibit anti-inflammatory properties by stabilizing protein conformation and preventing aggregation.

##### 3.4.2.2 Experimental findings

According to Table 2, the Au/Pt/Cu nanofluid demonstrated a significant ability to inhibit heat-induced BSA denaturation. The calculated IC_50_ value was 14.88 ± 0.09 µM, based on triplicate measurements within the tested concentration range of 12.5–200 µg mL^−1^. This result indicates that nanofluid provides measurable protection against protein denaturation under thermal stress.^75^ For comparison, the reference non-steroidal anti-inflammatory drug diclofenac sodium exhibited an IC_50_ value of 6.44 ± 0.02 µM, reflecting its higher potency as a clinically established anti-inflammatory agent (Fig. 9).

**Table 2:** Anti-inflammatory activity of the compounds

S. No.	Compounds	IC_50_ (µM ± SD)
1	Au/Pt/Cu	14.88 ± 0.09
2	Diclofenac sodium	6.44 ± 0.02

**Fig. 9:**
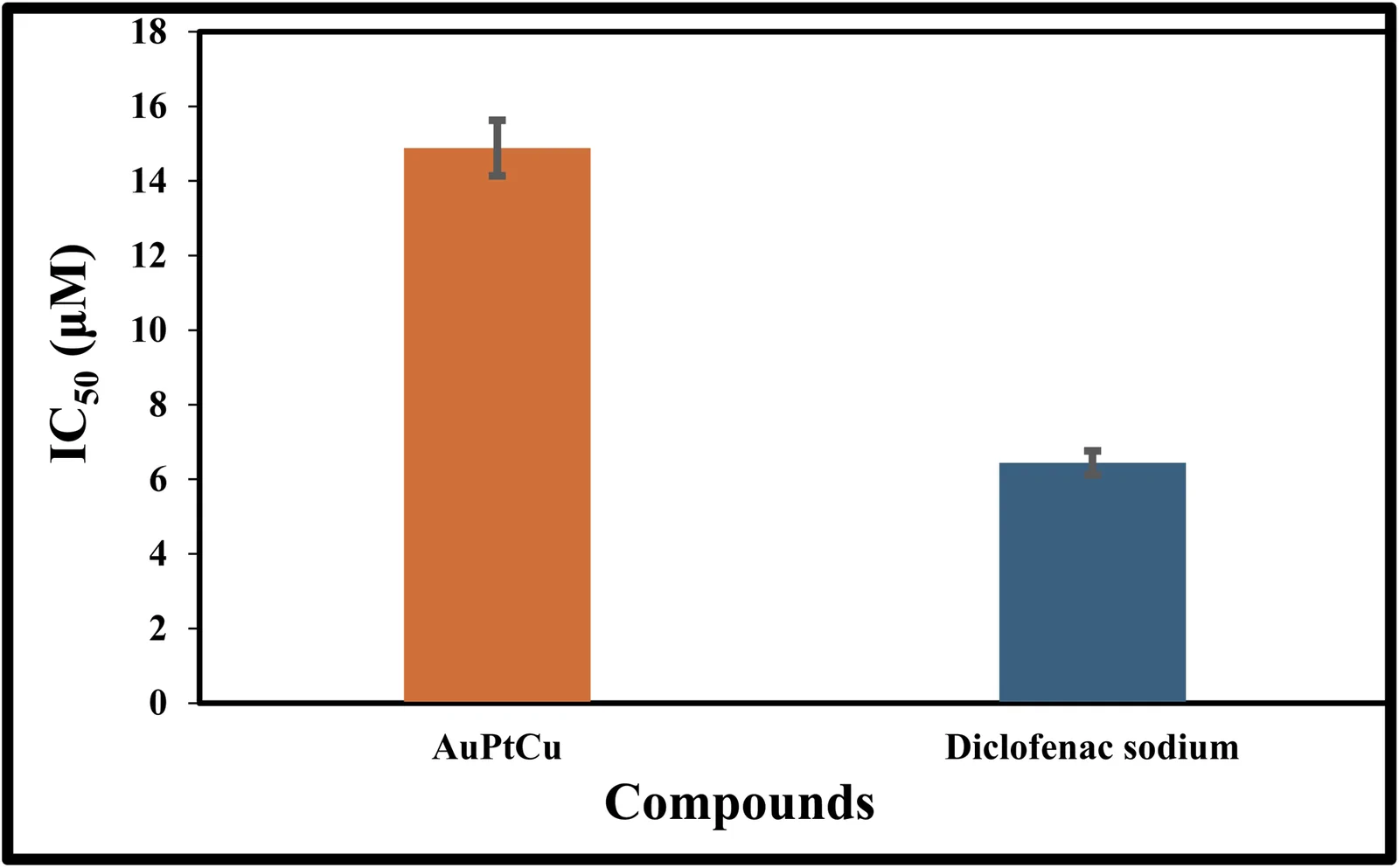
Anti-inflammatory activity of the Au/Pt/Cu.

##### 3.4.2.3 Mechanistic considerations

In addition to direct stabilization of proteins, multi metallic nanoparticles may modulate inflammatory responses through indirect mechanisms. For example, their ability to regulate ROS levels could reduce oxidative stress signals that trigger inflammatory cascades. Furthermore, stabilization of lysosomal membranes may limit the release of enzymes and mediators that contribute to inflammatory damage.

Future studies should explore these mechanisms using cellular models such as lipopolysaccharide-stimulated macrophages. Such experiments could measure the effects of the nanofluid on pro-inflammatory cytokines including TNF-α, IL-1β, and IL-6, as well as on molecular pathways involving COX-2, prostaglandin E_2_, and NF-κB activation. Assessment of hemocompatibility and complement system activation will also be necessary to evaluate its safety for biomedical use.

#### 3.4.3 Antioxidant activity

##### 3.4.3.1 Assay basis

The antioxidant properties of the trimetallic nanofluid were investigated using the DPPH radical scavenging assay. This widely used method evaluates the ability of compounds to neutralize a stable free radical through electron transfer or hydrogen atom donation. A reduction in the characteristic purple colour of DPPH indicates radical scavenging activity. Within the Au/Pt/Cu nanofluid, each metal contributes to the overall redox functionality. Gold facilitates efficient electron transfer processes, platinum provides catalytic sites capable of promoting peroxide transformations, and copper readily undergoes reversible redox cycling between Cu^+^ and Cu^2+^ states. The synergistic interaction of these metals may therefore enhance the radical-scavenging efficiency of the nanofluid.

##### 3.4.3.2 Antioxidant activity results

According to Table 3, the Au/Pt/Cu nanofluid exhibited strong antioxidant activity, with an IC_50_ value of 4.34 ± 0.08 µM obtained from triplicate experiments. This value indicates substantial radical-scavenging ability within the low-micromolar concentration range. As expected, the standard antioxidant ascorbic acid displayed greater potency, with an IC_50_ value of 1.95 ± 0.05 µM (Fig. 10). Nevertheless, the activity observed for the trimetallic nanofluid remains significant and reflects an effective capacity to neutralize free radicals in this chemical assay system.^76–78^

**Table 3:** Antioxidant activity of Au/Pt/Cu

S. No.	Compounds	IC_50_ (µM ± SD)
1	AuPtCu	4.34 ± 0.08
2	Ascorbic acid	1.95 ± 0.05

**Fig. 10:**
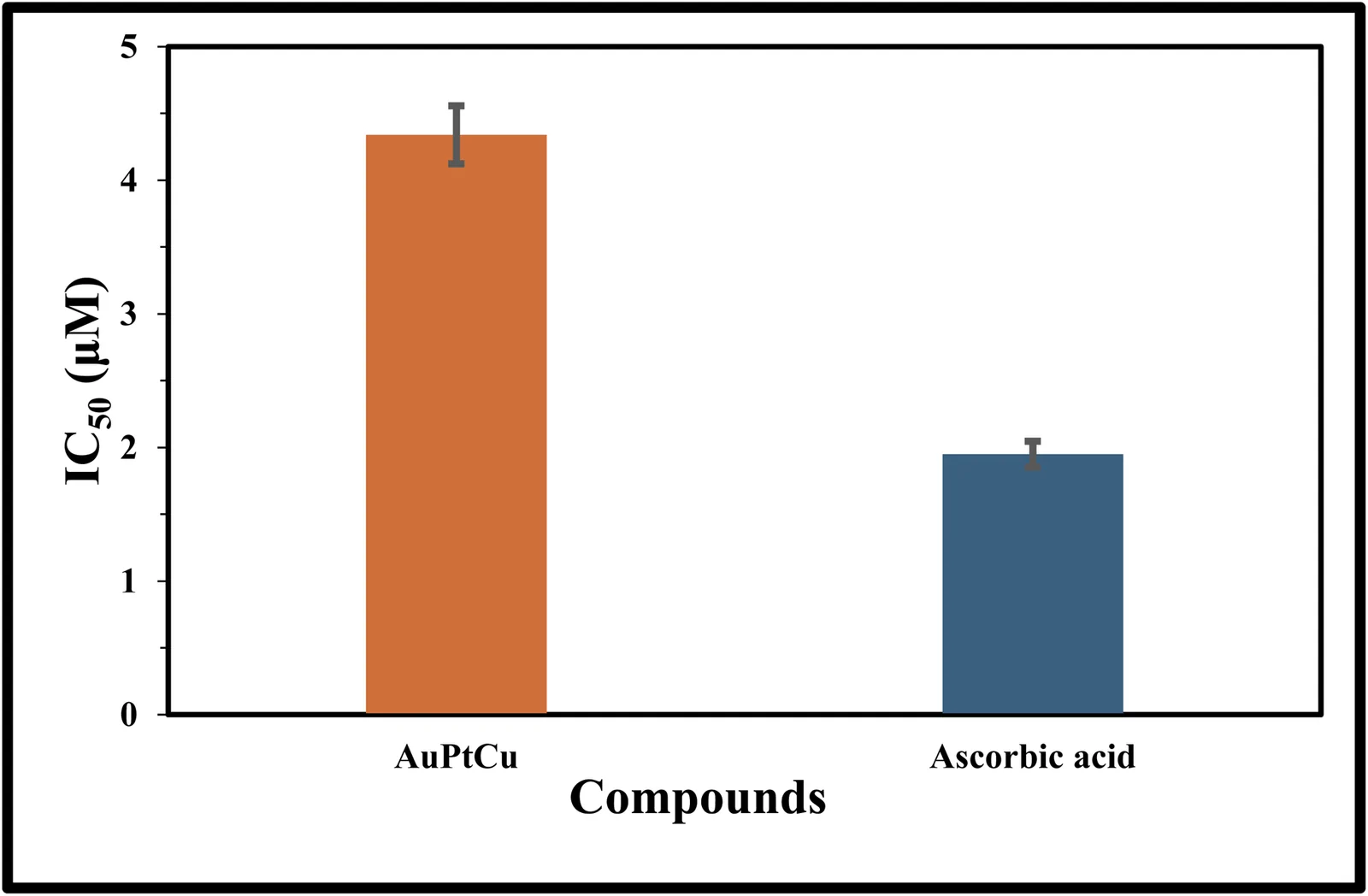
Antioxidant activity of the Au/Pt/Cu.

##### 3.4.3.3 Further evaluation

Additional chemical assays such as ABTS and FRAP would help assess whether the observed response is assay-dependent. Cell-based measurements using ROS-sensitive probes, mitochondrial superoxide assays, and lipid-peroxidation markers would be needed to evaluate intracellular redox effects.

#### 3.4.4 Integrative perspective

Taken together, the findings of this study demonstrate that the Au/Pt/Cu trimetallic nanofluid possesses a multifunctional biomedical profile characterized by antimicrobial activity, anti-inflammatory potential, and significant antioxidant capacity. The simultaneous presence of these properties suggests that the nanofluid could be particularly useful in clinical scenarios where infection, inflammation, and oxidative stress occur concurrently. Before translation toward biomedical applications, several important aspects should be systematically evaluated. These include cytotoxicity testing on relevant human cell lines to establish biocompatibility, as well as hemocompatibility assessments to determine interactions with blood components. Pharmacokinetic and biodistribution studies will also be required to understand how the nanofluid behaves within biological systems. *In vivo* studies involving infection or wound-healing models would provide valuable insight into therapeutic efficacy, particularly in environments where fungal pathogens contribute to disease progression. Additionally, formulation research focusing on stable delivery systems such as antimicrobial coatings, hydrogel matrices, or sustained-release depot formulations may facilitate practical biomedical implementation. Through such investigations, the therapeutic potential of the Au/Pt/Cu nanofluid can be more clearly defined for applications including antifungal coatings, anti-biofilm materials, and wound-care technologies designed to mitigate oxidative stress and inflammation.

### 3.5 DFT calculations

#### 3.5.1 Structural configuration, spin state selection, and geometric analysis

Experimental characterization using Energy Dispersive X-ray Spectroscopy (EDX) indicated an overall atomic ratio of Au : Pt : Cu ≈ 1.1 : 1 : 2.47. Based on this composition, two plausible cluster models, Au_2_Pt_2_Cu_5_ and Au_3_Pt_3_Cu_7_, were constructed and evaluated to identify the most representative structural configuration. Geometry optimization at the chosen level of theory revealed that the 13-atom Au_3_Pt_3_Cu_7_ cluster exhibits the lowest electronic energy and is therefore considered the most stable model. The electronic ground state of the selected cluster was further established by comparing different spin multiplicities. Among the tested states, the singlet configuration was found to be energetically more favorable than the triplet state. Consequently, all subsequent calculations were performed using the singlet spin state.

The fully optimized Au_3_Pt_3_Cu_7_ cluster adopts a compact and highly packed geometry corresponding to the global minimum on the potential energy surface. The structure shows significant atomic mixing, with Au (yellow), Pt (grey), and Cu (orange) atoms distributed throughout the cluster rather than forming segregated domains as shown in Fig. 11. This indicates strong heterometallic interactions and contributes to the overall stabilization of the nanoparticle.

**Fig. 11:**
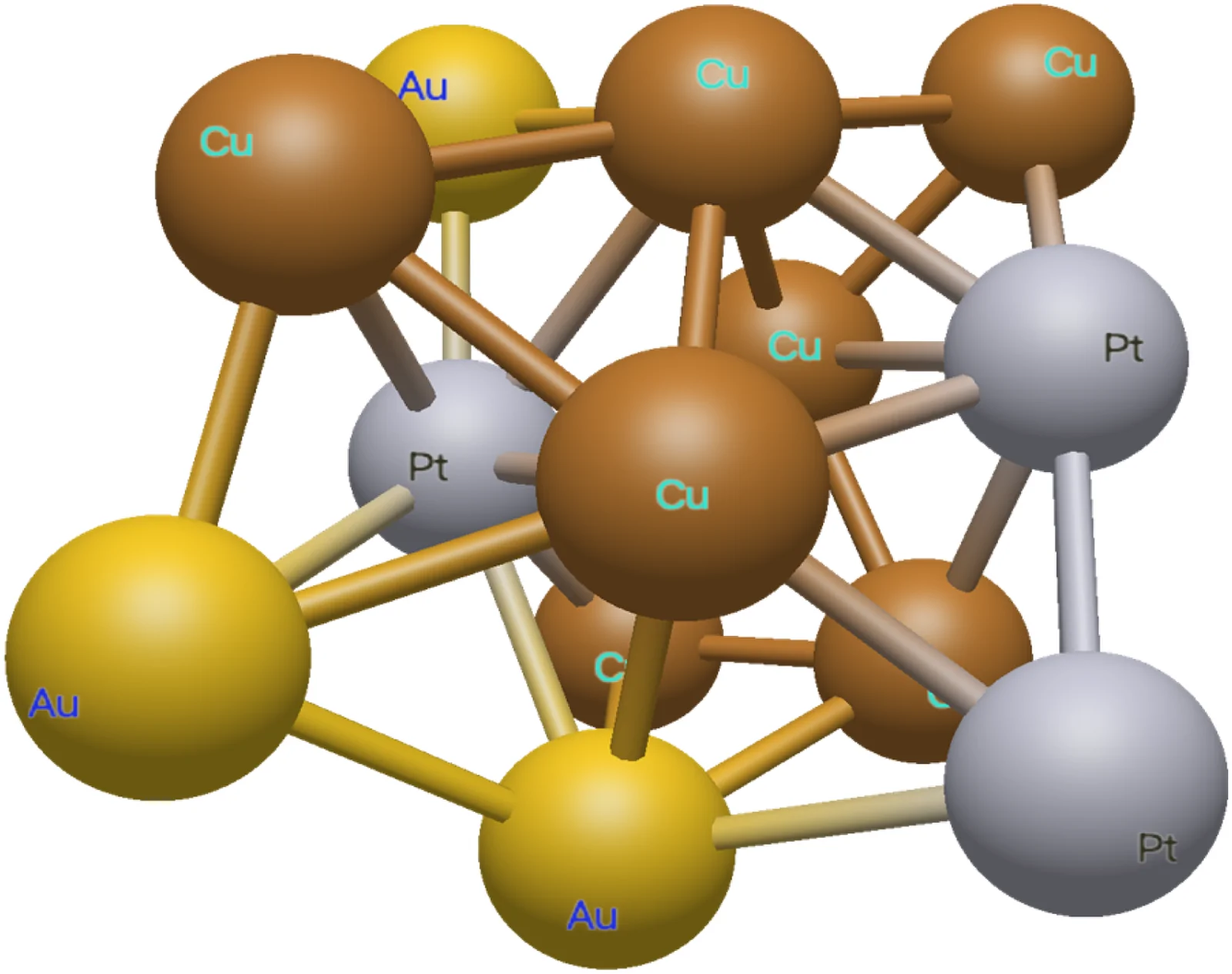
The optimized geometric structure of the 13-atom tri-metallic cluster Au_3_Pt_3_Cu_7._

#### 3.5.2 Multipole moments and polarizability analysis

The spatial distribution of electronic charge and its response to external electric fields were investigated through multipole moment analysis and evaluation of the static polarizability tensor. These properties provide important insight into the charge asymmetry and electronic flexibility of the cluster. The calculated total dipole moment of the Au_3_Pt_3_Cu_7_ cluster is 2.17 Debye, indicating a moderately asymmetric charge distribution arising from the heterometallic nature and non-uniform atomic arrangement of the system.^79,80^ Such a finite dipole moment suggests the presence of charge separation within the cluster, which may influence its interaction with external fields, surrounding media. The Heatmaps illustrate the atomic contributions to the dipole (*µ*), quadrupole (*Q*), and polarizability (*α*) tensor components of the Au_3_Pt_3_Cu_7_ cluster are shown in Fig. 12. The maps highlight the distribution and magnitude of individual atomic contributions along different cartesian directions, revealing the anisotropic nature of charge distribution and electronic response within the cluster. The dipole component analysis shows significant contributions along all cartesian directions, with net components of *µ*_*X*_ = −0.5733, *µ*_*Y*_ = 0.4303, and *µ*_*Z*_ = −0.4637 a.u. Quadrupole tensor components are predominantly negative along the diagonal directions (*Q*_*XX*_ = −192.6390, *Q*_*YY*_ = −177.4659, *Q*_*ZZ*_ = −177.9000), suggesting an overall contracted charge distribution, while off-diagonal terms remain relatively small. The polarizability tensor reveals high electronic deformability, with diagonal components *α*_*XX*_ = 413.9745, *α*_*YY*_ = 412.3410, and *α*_*ZZ*_ = 387.1389 a.u., indicating near isotropic but slightly anisotropic response (Table 4). Off-diagonal components are comparatively smaller, confirming that the primary polarization occurs along principal molecular axes.

**Fig. 12:**
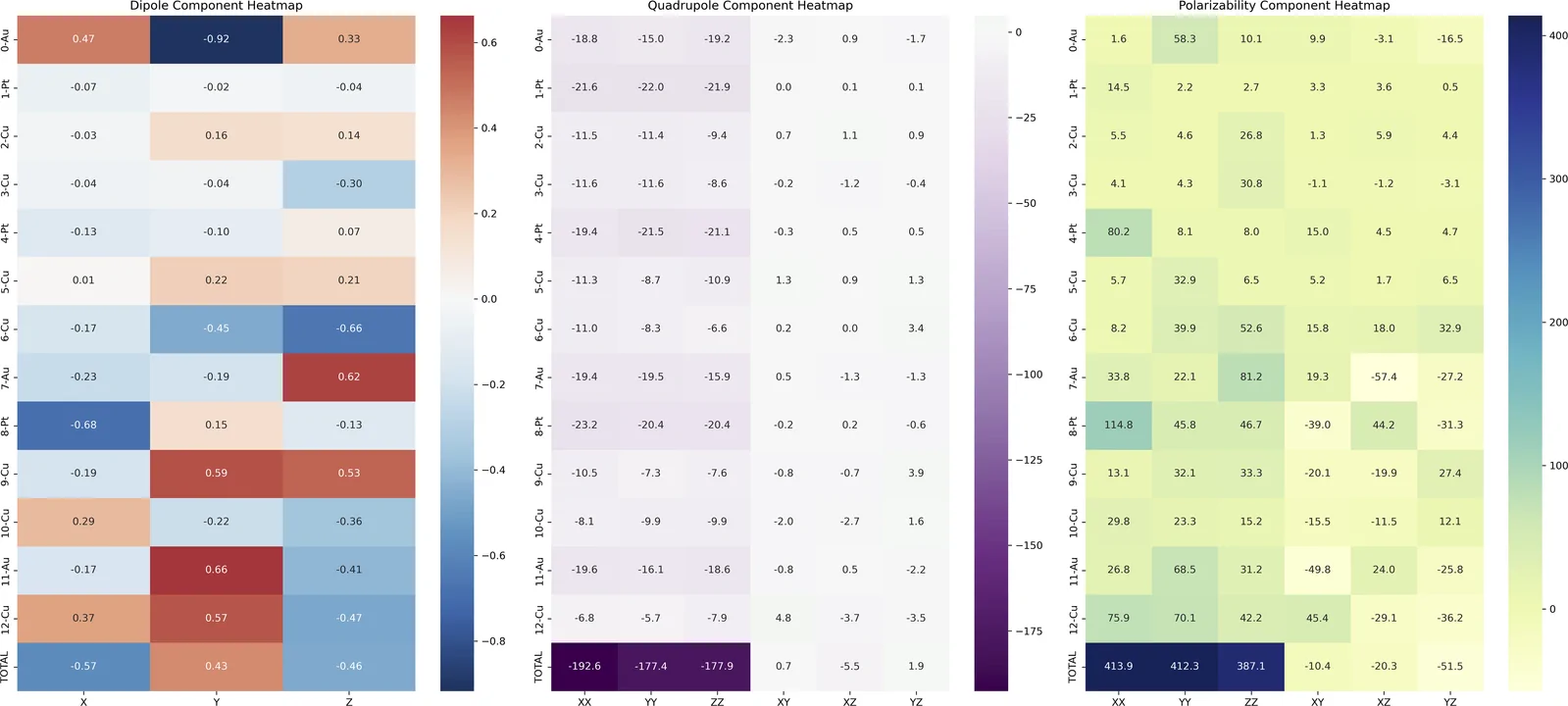
Heatmaps of atomic contributions to the dipole, quadrupole, and polarizability components of the Au_3_Pt_3_Cu_7_ cluster.

**Table 4:** Total multipole moments and polarizability for Au_3_Pt_3_Cu_7_

Sr. No.	Property (a.u.)	*X*/*XX*	*Y*/*YY*	*Z*/*ZZ*
1	Dipole moment	−0.5733	0.4303	−0.4637
2	Quadrupole moment	−192.6390	−177.3659	−177.9000
3	Polarizability	413.8745	412.3410	387.1389

#### 3.5.3 Vibrational spectroscopic data(s)

The calculated infrared (IR) and Raman spectra provide detailed insight into the vibrational characteristics of the Au_3_Pt_3_Cu_7_ cluster. For this 13-atom system, a total of 39 normal modes are expected, of which 33 correspond to genuine vibrational modes after excluding translational and rotational degrees of freedom. The vibrational frequencies are distributed over the range of 34–243 cm^−1^, which is characteristic of metal–metal bonded clusters as shown in Fig. 13. The low-frequency region (30–80 cm^−1^) is dominated by collective motions involving the heavier Au atoms, as well as global breathing modes of the cluster. These modes exhibit weak IR activity but moderate Raman intensity due to small changes in dipole moments and relatively larger changes in polarizability. In the intermediate region (80–150 cm^−1^), mixed vibrational modes involving Pt and Cu atoms are observed. These modes show enhanced IR intensity, primarily arising from asymmetric distortions within the cluster framework. A notable IR-active band at 136.37 cm^−1^ is assigned to asymmetric stretching modes involving significant Cu participation. The higher-frequency region (150–250 cm^−1^) is associated with more localized metal–metal stretching vibrations, predominantly involving Cu–Cu and Cu–Pt interactions. The Raman spectrum is characterized by a strong and intense peak near 187 cm^−1^, which can be attributed to symmetric metal–metal stretching vibrations accompanied by substantial changes in polarizability. The IR-active modes mainly originate from asymmetric vibrations that induce changes in dipole moment, whereas Raman-active modes are dominated by symmetric vibrations associated with variations in polarizability. This clearly demonstrates the complementary nature of IR and Raman spectroscopic techniques in probing the vibrational behavior of the cluster.

**Fig. 13:**
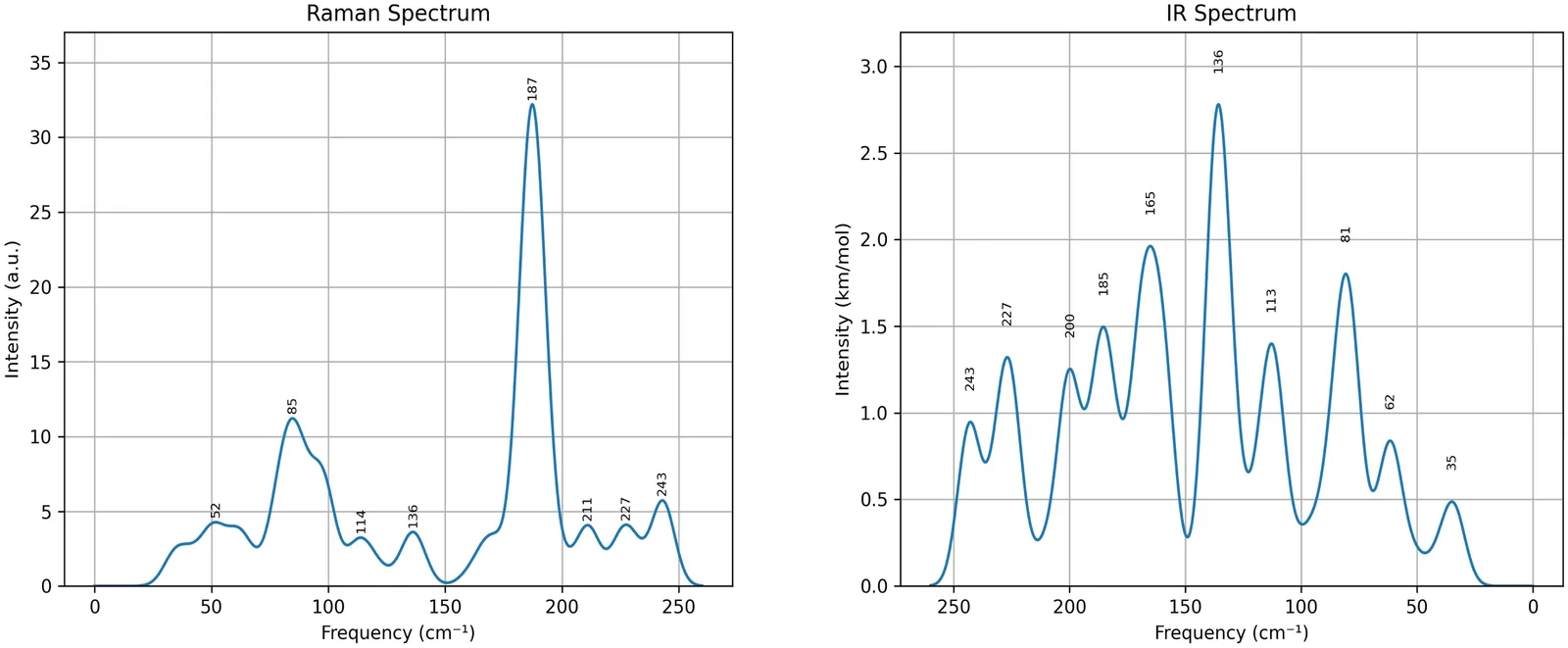
The calculated Raman spectrum and IR spectrum of Au_3_Pt_3_Cu_7_ cluster.

#### 3.5.4 Electronic properties, FMO analysis and thermochemistry

Frontier molecular orbital (FMO) analysis provides important insight into the electronic structure and reactivity of the Au_3_Pt_3_Cu_7_ cluster. The highest occupied molecular orbital (HOMO) is located at −5.2780 eV, while the lowest unoccupied molecular orbital (LUMO) lies at −3.2503 eV, resulting in a HOMO–LUMO energy gap of 2.028 eV (Fig. 14). This moderate energy gap suggests a favorable balance between electronic stability and chemical reactivity. The HOMO is associated with electron-rich regions of the cluster and represents electron donating sites, whereas the LUMO is localized over electron-deficient regions and represents electron-accepting sites. The spatial distribution of these FMOs identifies potential sites for charge transfer and surface chemical interactions which are relevant to the intrinsic reactivity of the trimetallic nanofluid. The comparatively small band gap represents that electron excitation, and charge transfer can occur with small energy source indicating favorable electronic characteristics for catalytic processes. These results provide theoretical insight into the electronic behavior of the Au_3_Pt_3_Cu_7_ cluster and complement the experimental observations.

**Fig. 14:**
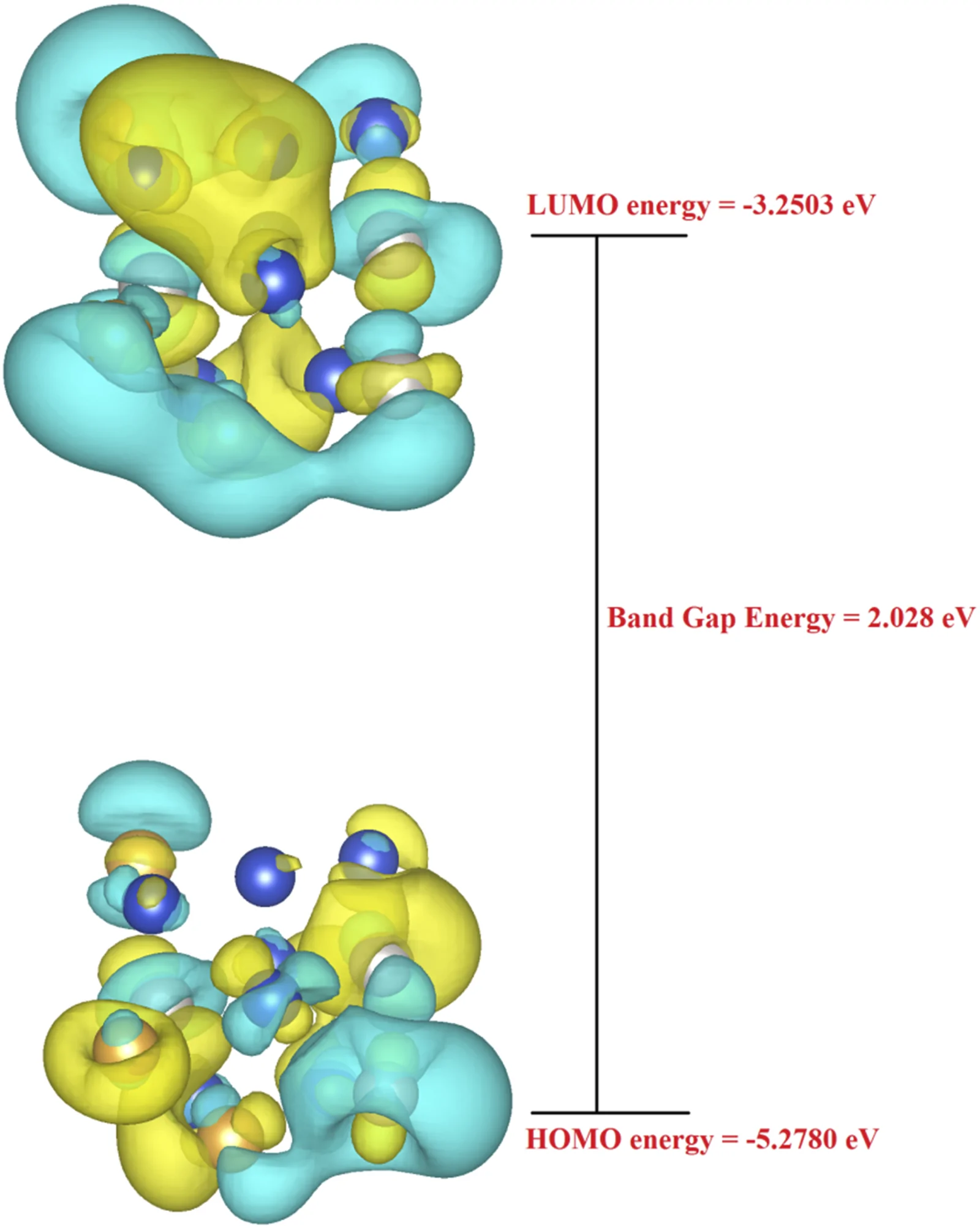
The optimized HOMO and LUMO energy levels of Au_3_Pt_3_Cu_7_.

Based on HOMO and LUMO energies, global reactivity descriptors were evaluated using Koopman's approximation.^81,82^ The ionization potential and electron affinity were calculated as 5.278 eV and 3.250 eV, respectively. From these values, the chemical hardness, softness, chemical potential, electronegativity, and electrophilicity index were derived as given in Table 5. Based on the calculated global reactivity descriptors, the molecule exhibits a balanced and biologically favorable electronic profile. The moderate chemical hardness (1.014 eV) together with relatively high softness (0.493 eV^−1^) suggests that the system possesses sufficient polarizability to interact effectively with biological targets such as enzymes and receptors. The negative chemical potential (−4.264 eV) indicates good thermodynamic stability while still allowing participation in charge-transfer processes. The relatively high electronegativity (4.264 eV) reflects a strong tendency to attract electrons, which may facilitate interactions with electron-rich biomolecular sites. Moreover, the significant electrophilicity index (8.96 eV) implies that the molecule can act as a potent electrophile, enhancing its ability to accept electron density from biological nucleophiles. This characteristic is often associated with improved binding affinity and potential inhibitory activity. The thermodynamic properties of the cluster were evaluated at 298.15 K. The electronic energy (*E*_el_) was calculated as −127 782.489 277 *E*_h_, while the zero-point energy (ZPE) is 0.009 183 *E*_h_ (5.76 kcal mol^−1^). The corrected enthalpy and Gibbs free energy were found to be −127 782.454 153 *E*_h_ and −127 782.538 913 *E*_h_, respectively. The large negative values of electronic and Gibbs free energy confirm the thermodynamic stability of the optimized Au_3_Pt_3_Cu_7_ cluster. Inclusion of zero-point and thermal corrections further refines the energetic profile, supporting the stability of the system under standard conditions.

**Table 5:** DFT calculations of electronic properties and global chemical reactivity descriptors of the Au_3_Pt_3_Cu_7_ nanofluid

Nano fluids cluster	Total energy (a.u.)	HOMO energy	LUMO energy	Band gap energy	Hardness (*η*)	Softness (*S*)	Electronegativity (*χ*)	Electrophilicity (*ω*)	Chemical potential (*µ*)
Au_3_Pt_3_Cu_7_	−127 782.4892	−5.2780 eV	−3.2503 eV	2.028 eV	1.014 eV	0.986 eV^−1^	4.264 eV	8.96 eV	−4.264 eV

The theoretical outcomes obtained from DFT calculations are in good agreement with the experimental characterization results. The optimized Au_3_Pt_3_Cu_7_ cluster model was established based on the EDX-derived atomic ratio, while the predicted heterometallic mixing and structural stabilization correlate well with the alloy/intermetallic phases and lattice distortions observed in XRD and HRTEM analyses. Furthermore, the moderate HOMO–LUMO energy gap and high electronic polarizability support the experimentally observed broad and red-shifted SPR absorption band in UV-vis spectroscopy, indicating enhanced electronic coupling and facile electron transfer within the trimetallic system. The calculated thermodynamic stability of the cluster is also consistent with the experimentally observed stable crystalline nanostructure.

## 4 Conclusion

Nanotechnology has garnered considerable attention recently for its potential applications across diverse fields. One notable area of exploration involves the development of nanofluid, offering innovative solutions with enhanced biological capabilities against a variety of ailments. The biological effectiveness of trimetallic Au/Pt/Cu nanofluid against numerous diseases can be attributed to several fundamental processes. Primarily, the nanoparticles possess a heightened surface area-to-volume ratio, facilitating increased contact with biological cells. This augmented interaction shows the disruption of the cell membrane, ultimately resulting in cellular demise. Additionally, nanoparticles interact with proteins, causing disruptions in their structure and functionality. This interference hinders vital cellular processes, culminating in the inhibition of infection growth and eventual cell death. Comprising copper, platinum and gold nanoparticles dispersed in a fluid medium, Au/Pt/Cu nanofluid presents a novel approach to combat microbial, inflammation and oxidant deformities. Notably, trimetallic Au/Pt/Cu nanofluid exhibit robust bioactivity, with a superior impact compared to both bimetallic and single metallic nanofluid. The disruption of pathogen cell walls, leading to increased permeability, contributes to the biological response of metal nanoparticles. The synergistic effects of constituent metals in bimetallic and trimetallic nanoparticles prove more effective in inducing noticeable changes in the cell membrane. In comparison to bimetallic and single metallic nanofluid, trimetallic nanofluid emerge as superior microbial, inflammation, oxidant inhibition agent. Moreover, Au/Pt/Cu nanoparticles-based nanofluid demonstrates enhanced biopotency against various microorganisms.

Overall, the trimetallic Au/Pt/Cu nanofluid exhibited promising antimicrobial, anti-inflammatory, and antioxidant activities, highlighting its potential for biomedical applications. Nonetheless, further research is imperative to comprehensively understand the action of mechanisms and potential applications of the nanoparticle.

The DFT studies on the Au_3_Pt_3_Cu_7_ cluster offer insight into the electronic structure and intrinsic reactivity of the Au/Pt/Cu alloy. The different findings including moderate HOMO–LUMO energy gap (2.028 eV), low chemical hardness, high softness, negative chemical potential and relatively high electrophilicity highlights promising electronic characteristics possessing good stability, polarizability and charge-transfer capability. These characteristics may encourage surface interactions and redox processes relevant to the experimental system. However, the observed antimicrobial, antifungal, antioxidant, and anti-inflammatory activities cannot be attributed solely to these global reactivity descriptors. Instead, they arise from the combined effects of the electronic structure, alloy composition, nanoparticle size, morphology, surface chemistry, and interactions with biological systems. Therefore, the DFT results provide qualitative theoretical support for the experimental findings rather than direct predictions of biological activity.

## Author contributions

Navneet Yadav: writing – original draft, methodology, data curation, visualization, investigation, formal analysis, review & editing, funding acquisition. Amara Rafi: formal analysis, modification, review & editing. Lalan Kumar: formal analysis (biomedical), modification, review & editing. Binesh Kumar: writing – original draft (biomedical), data curation, visualizations, validations, review & editing. Sikandar Paswan: writing – original draft, computational analysis, formal analysis, review & editing. Ajit Kumar Maddheshiya: formal analysis, review & editing. Jai Devi: formal analysis (biomedical), review & editing. Nikunj Patel: formal analysis (biomedical), review & editing. Afsanehsadat Larimi: formal analysis, review & editing. Bharat Kumar: writing – original draft, investigation, formal analysis, review & editing.

## Conflicts of interest

The authors declare that they have no known competing financial interests or personal relationships that could have appeared to influence the work reported in this paper.

## Data Availability

All data supporting the results of this article are provided in the file accompanying this manuscript. This includes all experimental datasets, characterization spectra, and relevant analysis data.

## References

[cit1] Sharma S., Chauhan A., Ranjan A., Mansor Mathkor D., Haque S., Ramniwas S., Singh Tuli H., Jindal T., Yadav V. (2024). Emerging challenges in antimicrobial resistance: implications for pathogenic microorganisms, novel antibiotics, and their impact on sustainability. Front. Microbiol..

[cit2] Waldman A. J., Balskus E. P. (2018). The human microbiota, infectious disease, and global health: challenges and opportunities. ACS Infect. Dis..

[cit3] Conte J. E. , Manual of Antibiotics and Infectious Diseases: Treatment and Prevention, Lippincott Williams & Wilkins, 2002

[cit4] Patel R., Patel N., Vegad K. (2024). Discriminating the leaves of Ocimum sanctum Linn. conferring to their age by gas chromatographic fingerprint analysis assisted with multivariate analysis. Trends Curr. Biol..

[cit5] Jiao Q., Zhi L., You B., Wang G., Wu N., Jia Y. (2024). Skin homeostasis: Mechanism and influencing factors. J. Cosmet. Dermatol..

[cit6] Ashley N. T., Weil Z. M., Nelson R. J. (2012). Inflammation: mechanisms, costs, and natural variation. Annu. Rev. Ecol. Evol. Syst..

[cit7] Liu J., Cao X. (2016). Cellular and molecular regulation of innate inflammatory responses. Cell. Mol. Immunol..

[cit8] Juan C. A., Manuel Pérez de la Lastra J., Plou F. J., Pérez-Lebeña E. (2021). The chemistry of reactive oxygen species (ROS) revisited: outlining their role in biological macromolecules (DNA, lipids and proteins) and induced pathologies. Int. J. Mol. Sci..

[cit9] Palengara D., Krishnan S., Eenthamvally S., Vadakkangara B. (2025). From invaders to innovators: biological potential of AgNPs biosynthesized using invasive alien plant species. Discov. Plants.

[cit10] Shivaramakrishnan B., Gurumurthy B., Balasubramanian A. (2017). Potential biomedical applications of metallic nanobiomaterials: a review. Int. J. Pharm. Sci. Res..

[cit11] Devi J. (2022). *et al.*, Synthesis, characterization, in vitro antimicrobial and cytotoxic studies of Co (II), Ni (II), Cu (II), and Zn (II) complexes obtained from Schiff base ligands of 1, 2, 3, 4-tetrahydro-naphthalen-1-ylamine. Appl. Organomet. Chem..

[cit12] Gawande M. B., Goswami A., Felpin F.-X., Asefa T., Huang X., Silva R., Zou X., Zboril R., Varma R. S. (2016). Cu and Cu-based nanoparticles: synthesis and applications in catalysis. Chem. Rev..

[cit13] Karimi-Maleh H., Karimi F., FallahShojaei A., Tabatabaeian K., Arshadi M., Rezapour M. (2019). Metal-based nanoparticles as conductive mediators in electrochemical sensors: a mini review. Curr. Anal. Chem..

[cit14] Tabassum H., Mahmood A., Zhu B., Liang Z., Zhong R., Guo S., Zou R. (2019). Recent advances in confining metal-based nanoparticles into carbon nanotubes for electrochemical energy conversion and storage devices. Energy Environ. Sci..

[cit15] Nwachukwu I. M., Nwanya A. C., Alshoaibi A., Awada C., Ekwealor A. B. C., Fabian I. E. (2023). Recent progress in green synthesized transition metal-based oxides in lithium-ion batteries as energy storage devices. Curr. Opin. Electrochem..

[cit16] Kim J. (2026). *et al.*, Engineering Biocompatible Metal-Based Nanoparticles for Advanced Antibacterial Therapy. ChemBioChem.

[cit17] Khalaf M. M. (2025). *et al.*, Innovative TiO 2/ZnO-organoselenium composites with diselenide linkages for enhanced anticancer efficacy. New J. Chem..

[cit18] Khalaf M. M. (2021). *et al.*, Crystalline Gold nanoparticles adjusted by carboxymethyl cellulose and citrate salt: Fabrication, characterization, and in vitro anticancer activity. J. Mol. Liq..

[cit19] Franco D., Calabrese G., Guglielmino S. P. P., Conoci S. (2022). Metal-based nanoparticles: antibacterial mechanisms and biomedical application. Microorganisms.

[cit20] Azharuddin M. (2019). *et al.*, A repertoire of biomedical applications of noble metal nanoparticles. Chem. Commun..

[cit21] Aswathy Waliaveettil F., Anila E. I. (2024). A comprehensive review on antibacterial, anti-inflammatory and analgesic properties of noble metal nanoparticles. Part. Part. Syst. Charact..

[cit22] Sharifi-Rad M. (2020). *et al.*, Green synthesis of silver nanoparticles using Astragalus tribuloides delile. root extract: Characterization, antioxidant, antibacterial, and anti-inflammatory activities. Nanomaterials.

[cit23] Solaiman M. A. (2020). *et al.*, Synthesis of Ag-NPs developed by green-chemically method and evaluation of antioxidant activities and anti-inflammatory of synthesized nanoparticles against LPS-induced NO in RAW 264.7 macrophages. Biocatal. Agric. Biotechnol..

[cit24] Kumar V. (2019). *et al.*, Green synthesis of silver nanoparticles using leaf extract of Holoptelea integrifolia and preliminary investigation of its antioxidant, anti-inflammatory, antidiabetic and antibacterial activities. J. Environ. Chem. Eng..

[cit25] Altalbawy F. M. (2026). *et al.*, Development and evaluation of a Zr-MOF/PVA nanofiber composite for efficient adsorption of malathion and carbofuran and antimicrobial control of aquatic pathogens. RSC Adv..

[cit26] Loza K., Heggen M., Epple M. (2020). Synthesis, structure, properties, and applications of bimetallic nanoparticles of noble metals. Adv. Funct. Mater..

[cit27] Crawley J. W. (2022). *et al.*, Heterogeneous trimetallic nanoparticles as catalysts. Chem. Rev..

[cit28] Liu L., Corma A. (2023). Bimetallic sites for catalysis: from binuclear metal sites to bimetallic nanoclusters and nanoparticles. Chem. Rev..

[cit29] Basavegowda N., Mandal T. K., Baek K.-H. (2020). Bimetallic and trimetallic nanoparticles for active food packaging applications: A review. Food Bioprocess Technol..

[cit30] Sathiyadevi G., Loganathan B., Karthikeyan B. (2014). Solvent-Mediated Eco-Friendly Synthesis and Characterization of Monodispersed Bimetallic Ag/Pd Nanocomposites for Sensing and Raman Scattering Applications. J. Nanosci..

[cit31] Iheme C. I. (2025). *et al.*, Eco-benign phytochemically-functionalized Ag-core Fe-shell bimetallic nanoparticles for targeted antioxidant and anti-inflammatory drug delivery. BMC Chem..

[cit32] Al-Sharqi A. A. (2026). *et al.*, Multifunctional Se–Cu bimetallic nanoparticles from marine Bacillus licheniformis: targeting oxidative stress, inflammation, and microbial biofilms. RSC Adv..

[cit33] Abirami P. (2025). *et al.*, One-pot synthesis of Ag-Cr bimetallic nanoparticles from Catharanthus roseus for anti-bacterial, anticancer, anti-diabetic, and anti-inflammatory activity and toxicity study in zebrafish. Biomass Convers. Biorefin..

[cit34] Zaleska-Medynska A., Marchelek M., Diak M., Grabowska E. (2016). Noblemetal-based bimetallic nanoparticles: the effect of the structure on the optical, catalytic and photocatalytic properties. Adv. Colloid Interface Sci..

[cit35] Long X. (2023). *et al.*, Capping and etching roles of copper ions in controlled synthesis of Au–PtCu trimetallic nanorods with improved photothermal and photocatalytic activities. Nanoscale.

[cit36] Gafner Y. (2018). *et al.*, Dual structural transition in small nanoparticles of Cu-Au alloy. J. Nanopart. Res..

[cit37] Li Z., Gao F., Zou B., Wu Z., Zhang Y., Du Y. (2021). Core@ shell PtAuAg@ PtAg hollow nanodendrites as effective electrocatalysts for methanol and ethylene glycol oxidation. Inorg. Chem..

[cit38] Hsu C. (2012). *et al.*, Synthesis of highly active and stable Au–PtCu core–shell nanoparticles for oxygen reduction reaction. Phys. Chem. Chem. Phys..

[cit39] Zhao Z. L. (2015). *et al.*, One-pot synthesis of small and uniform Au@ PtCu core–alloy shell nanoparticles as an efficient electrocatalyst for direct methanol fuel cells. Appl. Catal. B: Environ..

[cit40] Bian T. (2019). *et al.*, Seed-mediated synthesis of Au@ PtCu nanostars with rich twin defects as efficient and stable electrocatalysts for methanol oxidation reaction. RSC Adv..

[cit41] Rao K. J., Paria S. (2015). Mixed phytochemicals mediated synthesis of multifunctional Ag–Au–Pd nanoparticles for glucose oxidation and antimicrobial applications. ACS Appl. Mater. Interfaces.

[cit42] Karthikeyan B., Loganathan B. (2013). A close look of Au/Pt/Ag nanocomposites using SERS assisted with optical, electrochemical, spectral and theoretical methods. Phys. E: Low-Dimens. Syst. Nanostructures.

[cit43] Yadav N. (2018). *et al.*, Trimetallic Au/Pt/Ag based nanofluid for enhanced antibacterial response. Mater. Chem. Phys..

[cit44] Paul D., Mangla S., Neogi S. (2020). Antibacterial study of CuO-NiO-ZnO trimetallic oxide nanoparticle. Mater. Lett..

[cit45] Karrari S., Mohammadzadeh H., Jafari R. (2025). Characterization of ZnO-CuO and ZnO-CuO-NiO nanocomposites prepared by co-precipitation and antibacterial properties. Appl. Phys. A:Mater. Sci. Process..

[cit46] Alzahrani K. E. (2018). *et al.*, Antibacterial activity of trimetal (CuZnFe) oxide nanoparticles. Int. J. Nanomed..

[cit47] Hussein S. (2022). *et al.*, Green synthesis of trimetallic nanocomposite (Ru/Ag/Pd)-Np and its in vitro antimicrobial and anticancer activities. J. Chem..

[cit48] Ye X. (2019). *et al.*, One-pot synthesized Cu/Au/Pt trimetallic nanoparticles with enhanced catalytic and plasmonic properties as a universal platform for biosensing and cancer theranostics. Chem. Commun..

[cit49] Yuan Z. (2019). *et al.*, A trimetallic CuAuPd nanowire as a multifunctional nanocomposites applied to ultrasensitive electrochemical detection of Sema3E. Biosens. Bioelectron..

[cit50] He W. (2017). *et al.*, Formation of PtCuCo trimetallic nanostructures with enhanced catalytic and enzyme-like activities for biodetection. ACS Appl. Nano Mater..

[cit51] Song C. (2020). *et al.*, Colorimetric/SERS dual-mode detection of mercury ion via SERS-Active peroxidase-like Au@ AgPt NPs. Sens. Actuators, B.

[cit52] Nie F. (2020). *et al.*, Trimetallic PdCuAu nanoparticles for temperature sensing and fluorescence detection of H 2 O 2 and glucose. Front. Chem..

[cit53] Ding Y. (2018). *et al.*, FePt-Au ternary metallic nanoparticles with the enhanced peroxidase-like activity for ultrafast colorimetric detection of H2O2. Sens. Actuators, B.

[cit54] Pedone D., Moglianetti M., De Luca E., Bardi G., Paolo Pompa P. (2017). Platinum nanoparticles in nanobiomedicine. Chem. Soc. Rev..

[cit55] Su C., Huang K., Li H.-H., Lu Y.-G., Zheng Da-Li. (2020). Antibacterial properties of functionalized gold nanoparticles and their application in oral biology. J. Nanomater..

[cit56] Bogdanović U., Lazić V., Vodnik V., Budimir M., Marković Z., Dimitrijević S. (2014). Copper nanoparticles with high antimicrobial activity. Mater. Lett..

[cit57] Shaaban S. (2024). *et al.*, Synthesis, structural characterization, anticancer, antimicrobial, antioxidant, and computational assessments of Zinc (II), Iron (II), and Copper (II) chelates derived from selenated schiff base. Appl. Organomet. Chem..

[cit58] Shaaban S. (2025). *et al.*, Novel Fe (III), Cu (II), and Zn (II) Chelates of Organoselenium-Based Schiff Base: Design, Synthesis, Characterization, DFT, Anticancer, Antimicrobial, and Antioxidant Investigations. Appl. Organomet. Chem..

[cit59] Domingo L. R., Pérez P., Sáez J. A. (2013). Understanding the local reactivity in polar organic reactions through electrophilic and nucleophilic Parr functions. RSC Adv..

[cit60] Maddheshiya A. K. (2023). *et al.*, Facial synthesis of the trimetallic Au-Pt-Pd nanofluids and performance for methylene blue degradation. Mater. Lett..

[cit61] Loganathan B., Karthikeyan B. (2017). Tailored Au and Pt containing multi-metallic nanocomposites for a promising fuel cell reaction. J. Cluster Sci..

[cit62] Loganathan B. (2016). *et al.*, Tailored Rh surface facilitates, enhancement of Raman scattering in trimetallic AuPt core/Rh shell composites: Experimental and theoretical evidences. Phys. E: Low-Dimens. Syst. Nanostructures.

[cit63] Loganathan B. (2015). *et al.*, Synthesis and characterization of multimetallic-core and siliceous-shell Au/Pt/Ag@ SiO2 sol–gel derived nanocomposites. J. Sol-Gel Sci. Technol..

[cit64] Yadav N., Yadav R. R., Dey K. K. (2022). Microwave assisted formation of trimetallic AuPtCu nanoparticles from bimetallic nano-islands: why it is a superior new age biocidal agent compared to monometallic & bimetallic nanoparticles. J. Alloys Compd..

[cit65] Yadav N. (2020). *et al.*, Non-functionalized Au nanoparticles can act as high-performing humidity sensor. J. Mater. Sci.: Mater. Electron..

[cit66] Yadav N. (2026). *et al.*, Electrochemical performance of titanium (Ti), antimony-doped tin oxide on titanium (NiATOTi) and platinum-antimony-doped tin oxide on titanium (NiATOTiPt) anodes in closed loop flow wastewater treatment. Chem. Eng. J..

[cit67] Chaturvedi V. K. (2021). *et al.*, Two birds with one stone: oyster mushroom mediated bimetallic Au-Pt nanoparticles for agro-waste management and anticancer activity. Environ. Sci. Pollut. Res..

[cit68] Verma A. K. (2021). *et al.*, Study of Ultrasonic Attenuation and Thermal Conduction in Bimetallic Gold/Platinum Nanofluids: Effect of thermal conductivity on ultrasonic attenuation of gold and gold/platinum nanofluids. Johnson Matthey Technol. Rev..

[cit69] Singh Y. (2026). *et al.*, High-entropy oxide (CaCu3Ti3. 85Mn0. 05Sn0. 1O12) as a multifunctional electrocatalyst for supercapattery and water electrolysis. Nanoscale.

[cit70] Manikandan A., Sathiyabama M. (2015). Green synthesis of copper-chitosan nanoparticles and study of its antibacterial activity. J. Nanomed. Nanotechnol..

[cit71] Wang G. (2020). *et al.*, Effects of gold nanoparticle morphologies on interactions with proteins. Mater. Sci. Eng. C.

[cit72] Sangwan S., Seth R. (2022). Synthesis, Characterization and Stability of Gold Nanoparticles (AuNPs) in Different Buffer Systems. J. Cluster Sci..

[cit73] Dalal M. (2024). *et al.*, Unveiling the therapeutic potential of organotellurium (IV) Schiff base complexes through structural elucidation, antimalarial, antioxidant and antimicrobial studies. J. Mol. Struct..

[cit74] Arora T., Devi J., Rani M. (2024). Synthesis and characterization of indazole derived Schiff base ligands and their metal complexes: Biological and computational studies. Inorg. Chem. Commun..

[cit75] Khurana D. (2024). *et al.*, Unlocking the biological potential of transition metal complexes with Thiosemicarbazone ligands: Insights from computational studies. Heliyon.

[cit76] Taxak B. (2024). *et al.*, Hydrazone-containing organotin (IV) complexes: synthesis, characterization, antimicrobial, antioxidant activity and molecular-docking studies. BioMetals.

[cit77] Rani M. (2024). *et al.*, Unveiling Anti-malarial, Antimicrobial, Antioxidant Efficiency and Molecular Docking Study of Synthesized Transition Metal Complexes Derived From Heterocyclic Schiff Base Ligands. Chem. – Asian J..

[cit78] Kumar B., Khurana D., Devi J. (2024). Exploring antimalarial and antioxidant properties of hydrazone ligands and their transition metal complexes: Insights through molecular docking and ADMET studies. Inorg. Chem. Commun..

[cit79] Elking D. M., Lalith P., Robert D., Thomas D., Lee G. P. (2011). A finite field method for calculating molecular polarizability tensors for arbitrary multipole rank. J. Comput. Chem..

[cit80] Wang L., Michael S., David L. M., Christopher B., Michael K. G. (2024). A fast, convenient, polarizable electrostatic model for molecular dynamics. J. Chem. Theory Comput..

[cit81] Vijayaraj R., Subramanian V., Chattaraj P. K. (2009). Comparison of global reactivity descriptors calculated using various density functionals: a QSAR perspective. J. Chem. Theory Comput..

[cit82] andoval-Yanez C., Martínez-Araya J. I. (2019). Assessment of a set of twelve density functionals to estimate the global reactivity of myricetin through the Koopmans’ theorem. Chem. Phys. Lett..

